# Diet in Pregnancy: A Review of Current Challenges and Recommendations. A British Nutrition Foundation Briefing Paper

**DOI:** 10.1111/nbu.70016

**Published:** 2025-07-06

**Authors:** Kathryn H. Hart, Alyson J. Hill, Javier T. Gonzalez, Anne de la Hunty, Alison M. Gallagher, Sara A. Stanner

**Affiliations:** ^1^ Faculty of Health & Medical Sciences University of Surrey Guildford UK; ^2^ Nutrition Innovation Centre for Food & Health, School of Biomedical Sciences Ulster University Coleraine UK; ^3^ Centre for Nutrition, Exercise and Metabolism, Department for Health University of Bath Bath UK; ^4^ British Nutrition Foundation London UK

**Keywords:** birthweight, folic acid, maternal nutrition, micronutrients, pregnancy, pre‐pregnancy nutrition, weight gain

## Abstract

Pregnancy is a crucial period during which maternal nutrition, weight and lifestyle behaviours have a direct impact on both maternal and fetal health. This briefing paper describes dietary and lifestyle recommendations for women during the preconceptional period and throughout pregnancy, identifying specific factors that can be modified to improve health outcomes for both mother and child. It considers key areas such as nutrient intakes, supplementation, food safety and weight management, and highlights how dietary choices can help reduce the risk of common pregnancy‐related conditions. Despite widespread recognition of the importance of a healthy, balanced diet, many women in the UK fall short of recommended intakes for important nutrients, including iron, folate, iodine and vitamin D. These shortfalls are particularly evident among nutritionally vulnerable groups, such as teenagers, women from lower‐income households and those experiencing food insecurity; such groups may face barriers to accessing healthy foods and adhering to supplementation guidance. An increasing interest in plant‐based diets presents an opportunity to consider a range of dietary patterns that support both maternal health and environmental sustainability. However, such shifts must be carefully managed to ensure adequate intake of nutrients commonly found in animal products, such as vitamin B12, iron, iodine, calcium and long‐chain fatty acids. Rates of overweight and obesity among women of childbearing age remain high, reflecting trends in the general population and contributing to growing concern about maternal obesity. Maintaining a healthy weight before and during pregnancy plays a key role in supporting maternal and fetal wellbeing. Both insufficient and excessive weight gain are associated with elevated risks of complications. Excessive weight gain during pregnancy is associated with an increased risk of developing gestational diabetes, hypertensive disorders such as pre‐eclampsia, preterm birth and a greater likelihood of long‐term obesity in both mother and child. Supporting women to achieve and maintain a healthy weight in the periconceptional period and throughout pregnancy is therefore a public health priority. The antenatal period presents a unique window of opportunity to promote healthier and more sustainable eating patterns, as women are often highly motivated to improve their health and are in more regular contact with healthcare professionals at this time. Yet, research indicates that many women are unaware of dietary recommendations or receive inconsistent advice. To fully harness this opportunity, healthcare providers must be equipped with culturally appropriate, accessible and evidence‐based resources to support perinatal conversations around diet, supplementation, physical activity and body weight. Providing appropriate support during the periconceptional and early pregnancy period is essential to addressing health inequalities, improving long‐term wellbeing and positively influencing the health of future generations.

## 1 Introduction

Pregnancy is a significant life stage during which diet and nutrition profoundly influence both a mother's health and the development of her baby, with consequences that can extend across generations (British Nutrition Foundation 2013). During this period, a diet rich in essential nutrients is crucial for supporting fetal growth and preparing the mother's body for childbirth and lactation. Globally, there is growing recognition of the importance of a healthy, balanced diet before and during pregnancy, given its profound effects on both maternal and fetal health in the short and long term (Tuncalp et al. 2020).

This briefing paper builds on a previous briefing paper by the British Nutrition Foundation (BNF) (Williamson 2006) which examined physiological changes during pregnancy and the relationship between diet and pregnancy outcomes. It focuses on current UK dietary recommendations and specific nutrient concerns during pregnancy, with particular emphasis on the role of key nutrients and the role of supplements such as folic acid and vitamin D. In revisiting these topics, the review integrates new insights to better advise women during this crucial life phase.

A healthy diet before conception is vital for supporting fertility and preparing the body for pregnancy (Stephenson et al. 2018). Once pregnant, a woman's nutritional needs change significantly to support both the growing fetus and to maintain her own health. Key nutrients, including protein, complex carbohydrates, specific fatty acids and vitamins and minerals, must be consumed in appropriate quantities to ensure optimal fetal development and reduce the risk of pregnancy‐related complications.

Additionally, the briefing paper highlights the importance of achieving and maintaining a healthy weight before and during pregnancy, given its role in supporting both maternal and fetal health across the perinatal period and beyond. It also addresses safe food practices during pregnancy, emphasising the importance of food hygiene and highlighting certain foods that pregnant women in the United Kingdom are advised to avoid due to risks such as listeriosis and toxoplasmosis.

Acknowledging the health and environmental benefits of shifting towards more plant‐rich diets, the paper highlights the need to mitigate potential risks to the intake of important micronutrients, such as vitamin B12, iron, iodine and calcium, particularly for nutritionally vulnerable groups, while also addressing the socio‐economic inequalities that may affect access to nutritious food and supplementation.

By offering a comprehensive overview of these topics, this briefing paper aims to enhance understanding of the crucial role that diet plays in promoting a healthy pregnancy and ensuring positive birth outcomes in the United Kingdom.

Throughout this briefing paper, we have used the terms pregnant ‘woman’ or ‘mother’. These terms are intended to include all individual who are pregnant or have given birth, regardless of their gender identity.

## 2 Physiological Changes During Pregnancy

### 2.1 Weight Gain During Pregnancy

Weight gain during pregnancy is determined by the weight of the developing fetus and by increases in maternal tissues and results from the following components: fetus ~28%: fat stores ~26%: extracellular fluid ~9%: breasts ~8%: amniotic fluid ~8%: uterus ~8%; blood ~7%: placenta ~6% (Dalfra et al. 2022). Gestational weight gain (GWG) is therefore the total weight gained throughout pregnancy as a result of physiological changes (IOM 2009). Studies have shown that excess weight gained during pregnancy is associated with adverse fetal and maternal outcomes such as large for gestational age (LGA) baby, caesarean delivery and gestational diabetes mellitus (GDM), pre‐eclampsia and postpartum weight retention in the mother (Goldstein et al. 2017; Voerman et al. 2019). On the other hand, inadequate GWG has been linked to negative outcomes, including low birthweight (LBW) and small for gestational age (SGA) and an increased risk of preterm birth. Therefore, achieving appropriate GWG is crucial for an optimal outcome.

Currently, there are no recognised evidence‐based UK guidelines for weight gain during pregnancy and pregnant women are not routinely weighed unless there is a clinical reason to do so (e.g., having GDM). However, the National Institute for Health and Care Excellence (NICE) Guidelines on Maternal and Child Nutrition (NICE 2025) recommend that if people are interested in monitoring their weight change during pregnancy (e.g., if they have had excessive weight gain in a previous pregnancy), they should be referred to the US Institute of Medicine (IOM) (2009) guidelines for estimated healthy total weight change in singleton pregnancies (Table 1). These IOM guidelines recommend appropriate ranges of weight gain based on pre‐pregnancy body mass index (BMI), advising women entering pregnancy with a low BMI to gain more weight than women who are living with overweight or obesity. However, a key limitation of these guidelines is that they are not tailored to specific ethnic groups, which may reduce their applicability in ethnically diverse populations such as that of the United Kingdom.

**TABLE 1:** The US Institute of Medicine (IOM) recommendations for weight gain in pregnancy by pre‐pregnancy body mass index (BMI).

Preconception BMI	BMI (kg/m^2^) (WHO)	Total weight gain range (kg)	Rates of weight gain^a^ in the second and third trimesters (mean range in kg/week)
Underweight	< 18.5	12.7–18.1	0.45 (0.45–0.58)
Healthy weight	18.5–24.9	11.3–15.9	0.45 (0.36–0.45)
Overweight	25.0–29.9	6.8–11.3	0.27 (0.22–0.32)
Obesity (all classes)	≥ 30.0	5.0–9.1	0.22 (0.18–0.27)

*Note*: For twin pregnancy, the IOM recommends a GWG of 16.8–24.5 kg (37–54 lb) for women of normal weight, 14.1–22.7 kg (31–50 lb) for women living with overweight and 11.3–19.1 kg (25–42 lb) for women living with obesity. Modified from: Institute of Medicine and National Research Council (2009) (now the National Academy of Medicine (NAM)).

^a^ Calculations assume a 0.5–2.0 kg (1.1–4.4 lb) weight gain in the first trimester.

For women of healthy pre‐pregnancy bodyweight (BMI 18.5–24.9 kg/m^2^), the IOM (2009) recommended amount of weight gain during pregnancy is 11.3–15.9 kg. Within this range of weight gain, the risk for non‐elective caesarean delivery, postpartum weight retention, preterm birth, SGA or LGA birth and childhood obesity is minimised (IOM 2009). These weight gain recommendations include increases in fat stores and assume that women will breastfeed for at least 6 months, which has been shown to have its own positive health effects for both the mother and the infant (Victora et al. 2016).

### 2.2 Changes in Blood Composition

Increases in plasma volume can be detected as early as the first 6–8 weeks of pregnancy, reaching a peak increase of approximately 1500 mL by 34–36 weeks (Sanghavi and Rutherford 2014). This increase represents around a 50% increase in plasma volume when compared to an average, non‐pregnant European woman and tends to correlate more with fetal size than maternal body size. Red cell mass increases by around 250 mL, which reflects an 18% increase in the non‐pregnant red cell mass in women who do not supplement with iron but can increase by up to 400–450 mL in women who supplement with iron (Hytten 1985). Since plasma volume expands to a greater extent than red cell mass, haematocrit decreases. Nevertheless, the absolute increase in red cell mass increases the total oxygen carrying capacity, which can support the additional oxygen requirements demanded by the growing fetus, maternal organs and maternal activity. The increase in plasma volume can support the very large increases in blood flow to organs that require little additional oxygen, such as the skin and kidneys. These increases in blood flow are important for functions such as heat dissipation through the skin and enhanced renal filtration.

Plasma concentrations of metabolites, vitamins and hormones also change during pregnancy. Concentrations of lipids, fat‐soluble vitamins and specific carrier proteins typically increase, whereas concentrations of albumin, most amino acids, many minerals and water‐soluble vitamins tend to decrease during pregnancy. Increased glomerular filtration, which increases urinary excretion of some amino acids, vitamins and minerals, could explain part of the decrease in concentrations of these components. In general, however, decreased concentrations of nutrients are not necessarily indicative of altered nutritional status and could also be partly explained by plasma volume expansion.

### 2.3 Metabolic Changes and Adaptive Responses

Pregnancy results in a shift in metabolism, in large part due to changes in the concentrations of, and sensitivity to, various hormones. A physiological increase in insulin resistance (or decrease in insulin sensitivity) of maternal tissues is a normal response to pregnancy and results in a redirection of glucose from the mother to the fetus, ensuring a steady energy supply to support fetal growth and development. The fetus' heavier reliance on carbohydrates for fuel (Stander 1927) may relate to the lower requirement for oxygen in metabolising carbohydrate. Oxidising carbohydrate may therefore make the fetus more resilient to potentially fluctuating oxygen availability (Hooper 1995).

In normal pregnancy, insulin sensitivity is decreased and is accompanied by increased insulin secretion (Kampmann et al. 2019). This compensatory hyperinsulinaemia helps maintain normal maternal glucose levels despite rising insulin resistance. However, when insulin secretion does not adequately match the degree of insulin resistance, maternal blood glucose levels rise, potentially resulting in GDM. This can be influenced by a range of factors including pre‐existing insulin resistance (e.g., due to obesity, polycystic ovary syndrome or metabolic syndrome), genetic predisposition affecting beta‐cell function, age, ethnicity and lifestyle (Choudhury and Devi Rajeswari 2021).

The reduction in insulin sensitivity with pregnancy is thought to be due, in part, to changes in hormone availability from the placenta, such as human chorionic somatomammotropin (hCS), but the exact mechanism by which human pregnancy decreases insulin sensitivity is not fully characterised (Newbern and Freemark 2011). In addition to increasing glucose supply to the fetus, the decrease in insulin sensitivity increases fetal amino acid availability and contributes to the conservation of maternal lean tissue mass. The rate of nitrogen accumulation increases around 10‐fold over the course of pregnancy, with no apparent change in nitrogen balance of the mother (once accounting for nitrogen retention of the fetus and placenta). There is some evidence that urea synthesis and plasma urea concentrations are reduced in the last trimester. This suggests that maternal amino acid breakdown may be suppressed, possibly reflecting protein sparing (Elango and Ball 2016). However, previous studies of nitrogen balance may underestimate protein requirements, since more recent estimates of protein requirements using the indicator amino acid oxidation technique suggest that mean protein requirements may be 1.2 and 1.52 g/kg/day in early and late gestation, respectively (Elango et al. 2010) (compared to 0.75 g/kg/day for non‐pregnant women).

Other adaptive responses can assist in meeting increased nutrient demands irrespective of the nutrient status of the mother (Overduin et al. 2024). These include increased intestinal absorption of iron and calcium and potentially copper and zinc. Decreased urinary excretion of some micronutrients, such as riboflavin and taurine, has also been documented (Naismith et al. 1987; EFSA Panel on Dietetic Products, Nutrition and Allergies 2017), and increased aldosterone secretion may increase renal sodium reabsorption encouraging fluid retention.

### 2.4 Key Points

- Weight gain during pregnancy results from both fetal growth and increases in maternal tissues, including fat stores. Excess or inadequate weight gain is linked to adverse pregnancy outcomes, high or low birth weight and preterm birth. Although there are no UK‐specific guidelines for pregnancy weight gain, the US IOM and National Research Council (IOM 2009) provide evidence‐based recommendations based on pre‐pregnancy BMI to which women can be referred if they are interested in monitoring their weight change. These recommendations emphasise the importance of appropriate weight gain for minimising risks and supporting maternal and infant health.
- Plasma volume increases significantly during pregnancy and is mostly associated with fetal size. Red cell mass also increases, enhancing the total oxygen‐carrying capacity to meet the needs of the fetus and maternal organs despite a decreased haematocrit.
- Concentrations of lipids, fat‐soluble vitamins and specific carrier proteins increase, while albumin, most amino acids, minerals and water‐soluble vitamins tend to decrease due to increased glomerular filtration and plasma volume expansion.
- Pregnancy induces an increase in insulin resistance, redirecting glucose to the fetus. This adaptive mechanism supports fetal growth but can lead to gestational diabetes if insulin secretion is inadequate.
- Increased intestinal absorption and decreased urinary excretion of certain micronutrients, along with hormonal changes, help meet the heightened nutrient demands of pregnancy.

## 3 Development of the Fetus

### 3.1 Factors Associated With Birthweight

Birthweight is an important measure of infant health, with both low and higher‐than‐average birthweight associated with increased infant mortality and morbidity. Over the last 30 years, birthweight has increased globally, partly due to the rising prevalence of maternal obesity and gestational diabetes mellitus (GDM) (McMurrugh et al. 2024). Differences in average birthweight are also observed between races and ethnicities (Morisaki et al. 2017).

In the United Kingdom, the optimal outcome of pregnancy is the delivery of a full‐term (37–41 weeks), healthy infant with a birthweight of 3.1–3.6 kg (WHO 1995). This birthweight range is associated with favourable maternal outcomes, including reduced risk of mortality and pregnancy complications and supports fetal health by lowering the risk of prenatal and perinatal morbidity and promoting appropriate growth and development (WHO 1995).

The birthweight of a newborn depends on the duration of the pregnancy (gestation) and fetal (intrauterine) growth, which is influenced by maternal factors such as parity, BMI, age, socio‐economic status, education, health conditions, ethnicity and exposure to toxins such as alcohol and smoking. Infants born prematurely (before 37 weeks of pregnancy) are generally of low birthweight (LBW); however, a full‐term infant may be born small if unable to grow properly in the uterus. Small for gestational age (SGA) is defined as birthweight below the 10th percentile of recommended sex‐specific birthweight (De Onis and Habicht 1996). Infants from multiple births also tend to be smaller than singleton infants. In countries such as the United Kingdom and the United States, the percentage of preterm births (< 37 weeks) has risen steadily over the last decade, mainly among infants born at 32–36 weeks gestation due to advances in medical technology and neonatal care (ONS 2024; March of Dimes 2024; Fuchs et al. 2018), as well as the potential influence of increasing maternal age (Fuchs et al. 2018). However, there has been no measurable change in preterm birth rates over the last decade at a global level (Ohuma et al. 2023).

Low birthweight (< 2.5 kg), caused by intrauterine growth restriction, prematurity or both, is associated with increased morbidity and mortality in both infancy and later life. According to the WHO, LBW infants are approximately 20 times more likely to die within the first year of life compared to infants born at a healthy weight (WHO 2024), partly due to heightened susceptibility to infections and nutritional challenges in infancy. LBW is also associated with poor cognitive development in early life and an increased risk of chronic diseases such as cardiovascular disease, obesity and type 2 diabetes in adult life (see Section 3.2).

In the United Kingdom, the overall percentage of preterm LBW infants increased from 7.4% in 2020 to 7.9% in 2022 (ONS 2024), after a decade of relative stability. The prevalence is higher among lower socio‐economic classes (Thomson et al. 2021) and some ethnic minorities. Higher‐than‐average birthweight is also associated with increased risks of infant mortality, as well as obesity in adulthood and obstetric complications such as caesarean section, birth trauma and higher rates of neonatal morbidity and mortality (McMurrugh et al. 2024). While there is no universally accepted definition, macrosomia is generally defined as birthweight over 4 kg, though a birthweight of 4.5 kg is another cutoff used in clinical practice and literature (McMurrugh et al. 2024). Large for gestational age defines fetal overgrowth in relation to gestation, typically above the 90th birthweight percentile, although this is not standardised (McMurrugh et al. 2024). In England, 9% of births during 2023 to 2024 were over 4 kg (NHS England Digital 2024a) and an increase in birthweight has been noted over the last 30 years in the United Kingdom (McMurrugh et al. 2024) (see Section 7.4).

As noted previously, insulin resistance naturally increases during pregnancy and maternal tissues become increasingly insensitive to insulin as gestation advances, with a 50%–60% decrease in insulin sensitivity both in women with normal glucose tolerance and in women with GDM (Catalano 2013). Many factors influence insulin resistance such as placental hormones, obesity, physical inactivity, an ‘unhealthy’ diet and genetic and epigenetic contributions (Catalano 2013). Maternal insulin resistance, a common cause of excessive intrauterine fetal growth, leads to increased glucose transport across the placenta, causing fetal hyperglycaemia and hyperinsulinemia, resulting in excessive fetal growth (McMurrugh et al. 2024). This mechanism is further exacerbated by obesity and excessive GWG, both of which are risk factors for fetal macrosomia.

### 3.2 The Importance of the First 1000 Days

Early life nutrition, particularly during the first 1000 days—from conception to a child's second birthday—has a profound impact on lifelong health. This period is crucial, as both overnutrition and undernutrition are associated with long‐term consequences, influencing future risk of chronic disease and mortality (Barker 1998; Godfrey and Barker 2001). The concept of the ‘fetal origins’ of adult disease, initially proposed by Barker et al. (1989), highlights the link between lower birth weight and increased risk of cardiovascular disease, hypertension, type 2 diabetes and other metabolic conditions in later life. This hypothesis has been supported by various natural experiments and longitudinal follow‐up studies, including those examining populations affected by famine. For instance, individuals conceived during the Dutch Hunger Winter (1944–1945) who were exposed to famine during early gestation exhibited higher risks of obesity, glucose intolerance, hypertension and coronary heart disease in adulthood (De Rooij et al. 2021). Children exposed to the Dutch Hunger Winter famine in infancy, as well as those who experienced the Leningrad Siege (Stanner et al. (1997); Stanner and Yudkin 2001) and the Great Chinese Famine (Liu et al. 2017), were also more likely to experience poor health outcomes in adulthood. UK Biobank data has also been used to compare people who were or were not exposed to the sugar rationing of World War II in the United Kingdom, reporting a protective effect of rationing on later development of type 2 diabetes and hypertension (Gracner et al. 2024).

Although the potential for confounding in these studies is clearly a limitation, such findings underscore the potential importance of maternal nutrition during pregnancy and the early postnatal period; a concept now expanded into the broader framework of the Developmental Origins of Health and Disease (DOHaD). DOHaD posits that the nutritional environment during the first 1000 days can programme long‐term health outcomes, with poor nutrition and/or excessive growth during this period being linked to a higher risk of chronic diseases later in life.

The mechanisms underlying these associations, while not yet fully understood, are believed to involve ‘fetal programming’ or ‘nutritional programming.’ Adverse conditions *in utero*, for example resulting in an alteration in nutrient supply at a critical period in early life, can permanently affect the development and function of organs and tissues (Godfrey and Barker 2000). It is thought epigenetic modifications, changes in metabolic processes and alterations in neuroendocrine functions are likely contributors to this susceptibility (Yu et al. 2013; Hajj et al. 2014; Vickers 2022). Epigenetic changes refer to modifications to DNA, for example, via methylation, that regulate whether genes are turned on or off but which, unlike genetic changes (mutations), do not alter the sequence of nucleic acids in the gene. Research has also explored how the maternal gut microbiome and its interaction with the gastrointestinal system might impact nutrient processing and absorption, further influencing fetal growth (Hoffman et al. 2017).

While initial research emphasised the effects of maternal and fetal undernutrition, increasing evidence also indicates that maternal obesity before and during pregnancy is associated with a range of poor cardiometabolic outcomes in offspring, including obesity (Zheng et al. 2018; Reynolds et al. 2013). Maternal obesity affects fetal metabolism and can alter the amount of nutrients and metabolites transferred from the placenta to the fetus (Parrettini et al. 2020). Studies of adopted children demonstrate a strong correlation between BMI and their biological parents' BMI, which could suggest either a programmed or a genetic effect (Silventoinen et al. 2010). The importance of the maternal environment is highlighted by studies of siblings born to mothers living with obesity who underwent bariatric surgery. Offspring born after the mother had surgery and became lean had reduced adiposity and improved insulin sensitivity compared to siblings born before surgery, when the mother was living with obesity (Smith et al. 2009). Similarly, siblings exposed to maternal GDM had an increased BMI and were at a higher risk of developing type 2 diabetes in later life (Dabelea et al. 2000). These sibling comparisons suggest that the maternal environment *in utero* programmes these effects, rather than them being solely the result of genetic and postnatal environmental factors (Dearden and Ozanne 2023).

In summary, the first 1000 days represent a pivotal window during which maternal nutrition has a lasting impact on an individual's health trajectory. As evidence continues to grow, it is clear that both undernutrition and overnutrition during this period are significant correlates of chronic diseases in later life (Hoffman et al. 2017). This has particularly important implications in low‐ and middle‐income countries undergoing dietary transitions, where the effects of poor early‐life nutrition may be compounded by the increasing prevalence of overweight and obesity in adulthood (Kiosia et al. 2024). Understanding and addressing the nutritional needs during this crucial period is essential for improving health outcomes across the lifespan.

### 3.3 Essential Fatty Acids and Pregnancy

**FIGURE 1:**
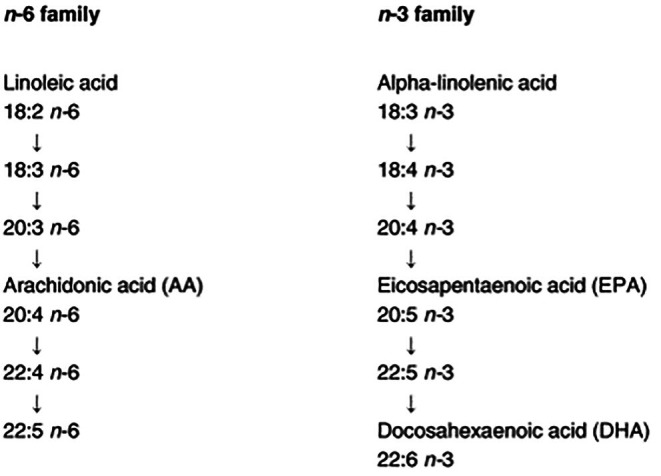
The *n*‐3 and *n*‐6 polyunsaturated fatty acid pathways. *Source*: Williamson (2006)

The essential fatty acids (EFAs), linoleic (LA, 18:2, *n*‐6) and α‐linolenic acid (ALA, 18:3, *n*‐3) and their related longer chain derivatives, arachidonic acid (AA, 20:4, *n*‐6), eicosapentaenoic acid (EPA, 20:5, *n*‐3) and docosahexaenoic acid (DHA, 22:6, *n*‐3), make an important contribution to the structure of cell membranes and are therefore essential to new tissue formation (Figure 1).

Dietary intake of these EFAs is important during pregnancy to support fetal development. Long‐chain polyunsaturated fatty acids (LC‐PUFAs), typically defined as fatty acids with 20 or more carbon atoms, are particularly critical for neural growth and development. An adequate supply of these LC‐PUFAs to the fetus is essential during pregnancy and can be met by maternal dietary intake, mobilisation of adipose tissue stores and/or *de novo* synthesis (Jones et al. 2014). Whether there are net benefits to taking LC‐PUFA supplements (e.g., fish oils) during pregnancy has been the topic of much discourse and the findings of research will be outlined in this section. It should also be noted that fish oil supplements generally contain purified *n*‐3 PUFAs, which distinguishes them from cod liver oil, which is rich in *n*‐3 PUFAs but also contains vitamin A and D. Due to these differences, it is recommended that pregnant women wishing to supplement with *n*‐3 PUFAs should do so with fish oils rather than cod liver oil, thereby avoiding excess intakes of vitamins A and D (see Section 6.1).

The long chain *n*‐3 fatty acid, DHA, can be synthesised from ALA by elongation and desaturation, but this synthesis is likely to be variable and limited. The most common dietary source of DHA is oily fish, which contain DHA from their consumption of DHA‐rich algae. In contrast to DHA, it is thought that the synthesis of AA from linoleic acid is sufficient to meet requirements (see Table 2 for typical dietary sources of EFAs). DHA is critically important for fetal brain and retina development (BNF 1999). DHA accumulates in high concentrations within retinal photoreceptor cells and is the most common fatty acid in brain cellular membranes. Together, DHA and AA constitute more than 30% of the brain and retinal phospholipids (Svennerholm 1964; Esmaeili et al. 2015).

**TABLE 2:** Dietary sources of *n*‐6 and *n*‐3 fatty acids.

		Food sources
*n*‐6 PUFAs	Linoleic acid	Vegetable/seed oils such as sunflower, corn, sesame, peanut and rapeseed and soybean oils and spreads made from these; nuts and seeds such as walnuts, brazil and pecan nuts, pine nuts, pumpkin and sunflower seeds and tahini paste
*n*‐3 PUFAs	Alpha‐linolenic acid	Some seeds (such as flax and chia seeds); walnuts and walnut oil, soya oil, rapeseed oil; meat from grass fed ruminants,^a^ as well as meat and eggs from animals fed an ALA enriched diet (e.g., labelled ‘omega‐3 enriched’ or ‘high in omega‐3’)
	EPA and DHA	Oily fish (e.g., salmon, trout, mackerel, sardines and herring); foods enriched or fortified with EPA/DHA

^a^ Studies suggest grass fed ruminants have higher omega‐3 fatty acid profiles compared to grain fed but these sources are considerably lower than quantities found in rich sources like oily fish.

Abbreviations: DHA, docosahexaenoic acid; EPA, eicosapentaenoic acid; PUFAs, polyunsaturated fatty acids.

The fetal brain grows most rapidly during the third trimester of pregnancy, which coincides with increased concentration of DHA in the fetal brain and retina during this time. Adequate dietary intake of long‐chain PUFAs is thus considered to be important during this time to support normal growth and neurological development and cognitive function.

To maintain EFA status during pregnancy, dietary EFA intake both before and during pregnancy must be sufficient to meet both maternal and fetal requirements. However, if dietary EFA intake is low, there is evidence that maternal stores of these fatty acids can be mobilised. Linoleic acid is found in high concentrations in adipose tissue, yet concentrations of adipose tissue ALA are much lower. The fatty acid profiles of an infant have been found to be highly correlated with that of its mother, suggesting that infant fatty acid status is possibly dependent on the maternal supply. During pregnancy, AA and DHA concentrations in maternal blood can fall by around 23% and 52%, respectively, by the time of birth. This is offset by an increase in concentrations of non‐essential fatty acids and normalisation post‐birth is relatively slow (Al et al. 1995).

When compared with singleton infants, infants of second and third pregnancies and those from multiple births have been shown to have lower DHA status. Infants born preterm have also been shown to have lower DHA status compared with those born full‐term. It has therefore been suggested that supplementation may be particularly important in cases of multiple births or closely spaced pregnancies, both of which can place greater demands on the maternal supply of long‐chain PUFAs to the fetus (Al et al. 2000).

There is evidence that supplementation with long‐chain *n*‐3 PUFAs during pregnancy may help to prevent premature delivery and low birth weight (Middleton et al. 2018). Several randomised controlled trials (RCTs) indicate that supplementation with DHA and EPA‐rich fish oils may modestly increase gestation length and/or birthweight. Meta‐analysis of 26 RCTs indicate that supplementation with *n*‐3 PUFAs via supplements and/or food can lower the risk of preterm birth (< 37 weeks) from 13.4% to 11.9%, representing a 0.89 (95% CI: 0.81–0.97) relative risk (RR) ratio (Middleton et al. 2018). Risk of LBW is also reduced from 15.6% to 14.0% (RR: 0.90, 95% CI: 0.82–0.99). There is also a suggestion of possible reduced risk of perinatal death (RR: 0.75, 95% CI: 0.54–1.03). There is currently insufficient evidence to determine the effects on maternal outcomes and child/adult offspring outcomes. The mechanism(s) responsible for these effects may include balancing of prostaglandins (mediators derived from PUFA) involved in the process of parturition or enhanced placental blood flow, potentially leading to increased fetal growth. It has also been suggested that long‐chain *n*‐3 PUFA supplementation could be particularly useful for preventing preterm delivery in high‐risk pregnancies (Allen and Harris 2001).

Public health guidelines around the world now consistently recommend that pregnant women consume fish and shellfish for general nutritional support (WHO 2016). Current consensus is that intakes of up to 1000 mg/day of DHA and EPA or up to 1000 mg/day of DHA alone, are not thought to raise safety concerns in the general population or pregnant women (Cetin et al. 2020) (but see Section 6.5 on advice around oily fish). It is desirable that pregnant women consume an additional intake of at least 100 to 200 mg/day of DHA (on top of the recommended minimum of 250 mg/day DHA + EPA combined) (EFSA 2010a), and that pregnant women with low DHA intake and/or low blood DHA status should receive a regular supply of around 600–1000 mg/day of DHA + EPA or DHA alone to reduce the risk of preterm birth (EFSA 2010a). This additional supply should preferably begin in the second trimester of pregnancy, not later than around 20 weeks of gestation, and should continue until 37 weeks if there is a risk of preterm birth. Women can be identified at increased risk of preterm birth due to low DHA intake and/or low status via dietary intake questions, which can be supported by the addition of a DHA measurement in blood. If considering supplements, caution is advised with respect to the potential for high vitamin A content of some fish oil supplements (e.g., cod liver oil, see Section 6.1).

### 3.4 Key Points

- Birthweight is a critical indicator of infant health, with both low and high birthweights linked to increased infant mortality and morbidity. Over the past 30 years, birthweights have risen globally due to factors such as maternal obesity and gestational diabetes.
- In countries such as the United Kingdom, a birthweight range for full‐term infants (37–41 weeks) of 3.1–3.6 kg is associated with favourable maternal and fetal outcomes. Birthweight is influenced by gestation length and maternal factors such as BMI, age, socio‐economic status, health conditions and exposure to toxins such as alcohol and smoking.
- Low birthweight infants (< 2.5 kg), often due to prematurity or intrauterine growth restriction, face higher risks of early mortality, infections, poor cognitive development and chronic diseases in adulthood. Conversely, higher‐than‐average birthweight (often defined as > 4 or > 4.5 kg) increases the risk of infant mortality, obesity in adulthood and obstetric complications such as caesarean section and birth trauma.
- The first 1000 days (from conception to age 2) are crucial for lifelong health. Undernutrition or overnutrition during this period can cause permanent changes in organ structure and function, increasing susceptibility to chronic diseases through mechanisms such as fetal programming and epigenetic modifications.
- Maternal obesity before and during pregnancy is also linked to adverse cardiometabolic outcomes in offspring, including increased risks of adiposity, type 2 diabetes and cardiovascular disease.
- The essential fatty acids (EFAs), linoleic acid (LA) and alpha‐linolenic acid (ALA), and their long chain derivatives such as arachidonic acid (AA) (derived from LA) and docosahexaenoic acid (DHA) (derived from ALA), are crucial for cell membrane structure and new tissue formation. DHA, derived from ALA, is vital for fetal brain and retina development, especially during the third trimester.
- Sufficient dietary intake of EFAs during pregnancy is critical to support both maternal and fetal needs. DHA is primarily obtained from oily fish and is necessary for neural growth and cognitive function. While AA synthesis from LA generally meets requirements, DHA synthesis from ALA can be variable and limited.
- RCTs suggest modest benefits of supplementation with long‐chain *n*‐3 PUFAs during pregnancy in extending gestation and increasing birth weight, alongside a potential reduction in preterm births and perinatal death risk. Public health guidelines recommend pregnant women consume additional DHA, with a suggested intake of 100–200 mg/day on top of the minimum 250 mg/day of DHA + EPA, preferably starting in the second trimester. However, pregnant women should avoid high vitamin A fish oil supplements such as cod liver oil.

## 4 Pre‐Pregnancy Nutritional Issues

### 4.1 Pre‐Pregnancy Weight, Fertility and Birth Outcome

It is accepted that fertility in women is affected by their percentage body fat, rather than absolute bodyweight. The Doubly Labelled Water sub‐study within the *National Diet and Nutrition Survey* (NDNS) reported that mean body fat for women aged 16–49 years was 37% ± 8% (Lennox et al. 2014), and, whilst an absolute cut‐off for fertility is debated, research has shown that a body fat content of 21%–22% may be necessary for the onset and maintenance of regular menstruation or the resumption of menses following Anorexia Nervosa‐related amenorrhea (Brage et al. 2020). Women who maintain a low bodyweight, who have suffered from eating disorders or who diet regularly often have irregular menstrual cycles and therefore may take longer to conceive (Goldberg 2002) and may also be more likely to suffer miscarriage or experience sexual dysfunction (Boutari et al. 2020). Gaining weight restores fertility, indicating that the relatively high percentage of body fat in females, compared with males, may influence reproduction directly. In an analysis of data from the *US National Health and Nutrition Examination Survey* (*NHANES*) study, each unit increase in BMI (when BMI was below 19.5 kg/m^2^) reduced the risk of infertility by 33% (Zhu et al. 2022).

However, excessive stores of body fat can also impair fertility—an estimated 3% increase in the risk of infertility for every unit increase in BMI above 19.5 kg/m^2^—and a substantial evidence base suggests impacts of obesity on all stages of conception via mechanisms including altered hormonal profiles and gonadotropin secretion and impaired embryo development and *in vitro* fertilisation outcomes, as well as a potential role in impaired oocyte quality and endometrial receptivity (Armstrong et al. 2022). In line with other health risks associated with central, as opposed to peripheral adiposity, the deposition of excess fat in central depots may also be more detrimental to fertility due to its role in driving insulin resistance and its effect on androgenic hormones and luteinising hormone, which may reduce egg viability.

A meta‐analysis of studies investigating the impact of lifestyle intervention on diagnosed infertility in women with a BMI over 25 kg/m^2^ reported beneficial effects on weight loss, ovulation incidence and sex hormone‐binding globulin levels, with an 11.23 times greater ovulatory incidence in intervention groups compared to controls (Sustarsic et al. 2023).

Women with a BMI ≥ 30 kg/m^2^ should be supported to reduce their weight before becoming pregnant, aiming for a loss of at least 5%–10% to improve their health and increase their chance of conception (NICE 2010). Further weight loss, to achieve a BMI within the healthy range (18.5–24.9 kg/m^2^) should also be encouraged, using evidence‐based behaviour change techniques. Conversely, low maternal BMI has been consistently associated with an increased risk of low birthweight infants and preterm births, but these women represent a heterogeneous group with respect to the underlying cause of their low weight and therefore the ability to mitigate against this with adequate GWG (Burnie et al. 2022) (see Section 3.1).

Having overweight or obesity prior to and during pregnancy is associated with an increased risk of several complications, including gestational diabetes, pregnancy‐induced hypertension, pre‐eclampsia and congenital defects, mediated in part through insulin resistance which, although common to all pregnancies, is exacerbated in mothers living with obesity. Obesity is also linked to a greater risk of complications during labour and to a greater risk of infants being large for gestational age or macrosomic. Furthermore, mothers with obesity during pregnancy are more likely to retain weight postpartum and have children who develop obesity (Williams et al. 2014).

As the prevalence of overweight and obesity is on the increase globally, such complications are a major cause for concern. Obesity is also the most common medical condition in women of reproductive age (Catalano and Shankar 2017), with over 20% of women in the United Kingdom entering pregnancy with obesity (RCOG 2018), an increase from 7% reported in 1990 (Heslehurst et al. 2010). Currently, 63% of women in Europe are living with overweight or obesity, including 35% who are specifically living with obesity (World Obesity 2024). Dieting to lose weight is not advisable during pregnancy (SACN 2024) (although gaining weight within the target weight gain recommendations is advisable to protect the health of mother and child). It is also important to note that certain weight loss drugs, particularly those that impact appetite or fat absorption, are contraindicated during pregnancy due to potential risks to both the mother and the developing fetus. Therefore, it is important that women who have overweight or obesity should attempt to reach a healthy bodyweight (i.e., a BMI of 20–25 kg/m^2^) before trying to conceive. Furthermore, preconception BMI has a greater influence on maternal health and the health of the fetus than the amount of weight gained during pregnancy (SACN 2024).

### 4.2 Pre‐Pregnancy Nutritional Status

It is now well established that maternal nutritional status at the time of conception is an important determinant of embryonic and fetal growth. The embryo is most vulnerable to the effects of poor maternal diet during the first few weeks of development, often before pregnancy has been confirmed. Cell differentiation is most rapid at this time and any abnormalities in cell division cannot be corrected at a later stage. Most organs, although very small, have already been formed 3–7 weeks after the last menstrual period and any teratogenic effects (including abnormal development) may have occurred by this time. Many women of childbearing age are not meeting their nutrient requirements (see Section 5) and improving nutritional status in women prior to pregnancy has a beneficial influence on subsequent birth outcomes (Stephenson et al. 2018). If all women of childbearing age were to consume a varied and adequate diet, this would help to correct the majority of nutritional imbalances and would help to ensure that, once pregnant, the fetus has the best nutritional environment in which to develop. Attention to the diet prior to pregnancy also sets appropriate dietary habits to be followed throughout pregnancy.

The NHS provides advice for women planning a pregnancy although this includes limited dietary advice, currently mentioning only the importance of folic acid supplementation (NHS 2023a) (see Section 4.2.2) and avoiding alcohol and certain contra‐indicated foods (see Section 6). Specific nutrients of concern in the pre‐conception period are addressed below but general healthy eating principles remain relevant, and it is prudent for women wishing to conceive to adopt pregnancy‐specific dietary recommendations with respect to caffeine, alcohol, vitamin A and fish (see Section 6).

#### 4.2.1 Vitamin D

Adequate vitamin D levels are vital for fetal bone development and immune function (see Section 5.5.5). Women in the United Kingdom, including those who are planning a pregnancy, are advised to consider taking a daily vitamin D supplement (10 μg/day) in the autumn and winter months (from October to March). Some ‘at risk’ groups may benefit from considering a supplement throughout the year, including those with darker skin (e.g., African, African Caribbean or South Asian origin) and those who cover their skin when outside or spend a lot of time indoors (NHS 2023b). In data collected between 2019 and 2023, the NDNS showed 26% of 11‐18 year‐old girls and 17% of adult women aged (19‐64 years) had low vitamin D status (defined as 25‐hydroxyvitamin D concentration <25 nmol/L) (OHID 2025) (Table 9).

#### 4.2.2 Folate/Folic Acid

It is well recognised that folic acid (the synthetic form of the vitamin folate) is of critical importance both pre‐ and peri‐conceptionally to protect against neural tube defects (NTDs) in the developing fetus. The neural tube develops into the spine and NTDs occur when the brain and skull and/or the spinal cord and its protective spinal column do not develop properly within the first 4 weeks after conception (Engelhardt et al. 2022). The most common NTDs are anencephaly, which results in stillbirth or death soon after delivery, and spina bifida, which may lead to a wide range of physical disabilities, including partial or total paralysis. Risk of congenital heart malformations and orofacial clefts, such as cleft lip and palate, has also been associated with periconceptual folic acid status (McNulty et al. 2023).

There are variations in the prevalence of NTDs which depend on demographic, ethnic and social factors. Environmental factors also seem to influence the risk. Between 2000 and 2019, there were 4541 NTD pregnancies (based on rates of anencephaly, spina bifida and encephalocele only) out of 3 637 842 births in England. There has been little change in NTD prevalence across Europe in recent years, with the data from England suggesting overall rates may even be increasing (Broughan et al. 2024). According to the latest NDNS (2019 to 2023), 83% of women aged 16–49 years in the UK had folate levels associated with an increased risk of NTDs, such as spina bifida (OHID 2025).

The seminal study linking folic acid supplementation to NTD risk was conducted by the MRC across seven countries. This multicentre, randomised, double‐blind, prevention trial established that folic acid supplementation (4 mg/day in high‐risk women) before conception had a 72% protective effect on the incidence of NTDs in pregnancy (MRC 1991). Wald et al. (2001), by collating the data from a series of published supplementation trials, subsequently established a clear dose–response relationship and specifically identified that a dose of 5 mg daily would reduce NTD risk by about 85%, with an increase of 0.4 mg/day (400 μg) associated with a 36% risk reduction.

Despite this, advice in the United Kingdom has remained unchanged since 1992, recommending that women who could become pregnant take a daily supplement of folic acid (400 μg) prior to conception and until the 12th week of pregnancy (Department of Health 1992). Women not taking folic acid at the point of conception should still be encouraged to start this as soon as possible before the 12th week. Folate remains important later in pregnancy but is required at a lower dose (see Section 5.5.2). Those women deemed as higher risk are recommended to take a higher dose (5 mg/day) which is only available on prescription; higher risk currently includes women who have had a previous NTD‐affected pregnancy, couples where either partner has a family history of congenital malformations, and women with type 1 or type 2 diabetes or those who have a haematological condition requiring folic acid supplementation or are taking medication that could interfere with folic acid absorption.

In all cases, supplementation is assumed to be in addition to, not instead of, usual dietary recommendations (200 μg/day for adults) and so folate‐rich foods (naturally occurring or fortified, see Table 3) should still be encouraged.

**TABLE 3:** Sources of key nutrients of relevance to pregnancy in the United Kingdom (per 100 g and per portion).

Food	Portion	Folate/folic acid (μg)		Iodine (μg)		Iron (mg)		Vitamin D (μg)	
		Per 100 g	Per portion	Per 100 g	Per portion	Per 100 g	Per portion	Per 100 g	Per portion
Cereals and cereal products									
Yeast extract	Thinly spread on bread (3 g)	2620	**79** ^(2)^	49	1.5	2.9	0.1	0	0
Fortified breakfast cereals^a^	30 g	191–255	57–76	Trace	0	11–14.7	**3.3–4.4** ^(1)^	0–4.7	0–1.4
Wholemeal bread	1 medium slice	40	14.8	0	0	2.4	0.9	0	0
White bread^b^	1 medium slice	25	9	4	1.4	1.6	0.6	0	0
Fruits and vegetables									
Apple, eating, raw (with skin)	1 medium (174 g)	Trace	0	4	7	0.1	0.2	0	0
Banana, flesh only	1 medium (100 g)	14	14	3	3	0	0	0	0
Raspberries, raw	53 g	55	29	4	2.1	0.4	0.2	0	0
Oranges, flesh only	Small/medium orange (162 g)	33	53	1	1.6	0.1	0.2	0	0
Orange juice, from concentrate	160 mL glass	18	29	2.2	3.5	0.1	0.1	0	0
Dried apricots	26 g	14	3.6	0	0	4.1	1.1	0	0
Kale, boiled	One cup, chopped (130 g)	97	**126** ^(1)^	2	2.6	1.1	1.4	0	0
Broccoli, boiled	85 g	34	28.9	2	1.7	0.6	0.5	0	0
Peas, frozen, boiled	Three tablespoons (80 g)	31	24.8	2	1.6	1.8	1.5	0	0
Lentils, red, dried, boiled	½ cup (96 g)	36	34.6	2	1.9	2.1	2.1	0	0
Peanuts, dry roasted or roasted and salted	10 kernels (13 g)	44–52	5.7–6.8	19	2.5	1.3–2.1	0.2–0.3	0	0
Meat, fish and alternatives									
Beef mince, cooked	Average portion (85 g)	5	4.3	14	11.9	2.7	**2.3** ^(2)^	0.4	0.3
Chicken breast, grilled without skin	Medium fillet (120 g)	6	7.2	7	8.4	0.4	0.5	0.3	0.4
White fish, for example, cod	Medium fillet (108 g)	8	8.6	161	**174** ^(1)^	0.15	0.2	Trace	< 0.01
Oily fish, for example, salmon	Average steak (210 g)	17.9	37.6	4.2	8.8	0.4	0.9	9.2	**19.2** ^(1)^
Eggs, boiled	1 average egg (50 g)	30	15	52	26	2	1.0	3.2	**1.6** ^(2)^
Dairy and alternatives									
Milk, semi‐skimmed	250 mL glass	9	23	30	**75** ^(2)^	0.02	0.05	Trace	< 0.01
Milk, plant based^c^	250 mL glass	0–30	0–75	0.05–22.3	0.1–56	0–0.4	0–1.1	0–1.5	0–3.8
Cheese, cheddar	Average grated portion (30 g)	31	9.3	30	9	0.3	0.1	0.3	0.1
Yogurt (fat free, low fat, whole milk)	Average pot (125 g)	18	22.5	34–63	43–79	0.1	0.1	0–0.1	0–0.1

*Note*: Figures in bold within a column represent first^(1)^ and second^(2)^ highest source per portion for the respective nutrient, NOT accounting for bioavailability.

^a^ Average of fortified corn flakes, wheat biscuits, chocolate flavoured rice and multigrain hoops.

^b^ From 2026, white flour will have to be fortified with folic acid (250 μg of folic acid per 100 g of non‐wholemeal flour).

^c^ Average of unsweetened soya, fortified oat and unsweetened and unfortified oat. Please note brands and sub‐types (organic, (un)sweetened, fortified) vary significantly so consumers are encouraged to check the label.

*Source*: McCance and Widdowson's The Composition of Foods 7th edition (Public Health England 2014), all rounded to 1 dp.

This emphasis on supplementation is due to the fact that the extra folate required peri‐conceptionally is difficult for women to obtain through the diet alone. Folic acid (the synthetic form) is also more bioavailable and stable than the natural folates found in food (FAO/WHO 2022), hence foods fortified with folic acid are also an important source.

Although there have been several government campaigns to increase awareness of the importance of folic acid supplementation, many pregnancies are still unplanned (PHE 2018)^1^ and in these cases, women often do not start taking folic acid supplements until the pregnancy has been confirmed. Studies attempting to quantify United Kingdom compliance with pre‐conceptual folic acid supplementation suggest, at best, only 1 in 2 women are complying (for those with a previous NTD‐affected pregnancy) (Bestwick et al. 2014) but at worst, there may be as few as 27.3% (Schoenaker et al. 2023) or 1 in 5 for some ethnic minority populations (Bestwick et al. 2014). There is evidence that the proportion of women supplementing has declined in recent years; 19.7% of women in England were taking folic acid supplements before pregnancy in 2023 to 2024 compared with 25.7% in 2019 to 2020 and this was lower in the most deprived areas (10.1%) compared with the least deprived (27.9%) (OHID 2024).

After over 20 years of evidence‐gathering and debate, and following recommendations from the Scientific Advisory Committee on Nutrition (SACN), the UK Government agreed in 2021 that there should be mandatory fortification of non‐wholemeal flour with folic acid (Department of Health 2021). Mandatory (as opposed to voluntary) folic acid fortification has been shown to be highly effective in increasing population folate status, with data from the US and Canada showing significant improvements in short‐ and long‐term biomarkers of status and associated decreases of between 27% and 50% in NTD rates (De Wals et al. 2007; Crider et al. 2018). Legislation announced in the United Kingdom in November 2024 will require millers and flour producers to fortify non‐wholemeal wheat flour with folic acid from the end of 2026. However, the policy has been criticised for not going far enough–since the level of fortification and the limited products included (white flour only as opposed to wholemeal flour and other grains) would only be expected to prevent 20% of the current annual cases of NTD (Looi 2023). There is also concern that it may discriminate against those who choose relatively healthier wholemeal products or non‐flour based starchy staples, as well as ethnic minorities, who do not consume non‐wholemeal flour as their staple carbohydrate or have a limited access to a varied diet due to cost. As such, continuing to promote a folate‐rich diet and greater uptake of pre‐conceptual supplementation remains a priority in the United Kingdom.

#### 4.2.3 Iodine

Following the publication of evidence by VanDer Pump et al. (2011) suggesting that the United Kingdom was ‘iodine deficient’, the Scientific Advisory Committee on Nutrition (SACN), which provides advice to the UK government on nutrition‐related matters, undertook a scoping review and published its ‘Statement on Iodine and Health’ in 2014 (SACN 2014). Reproduction and gestation are recognised as vulnerable periods for iodine insufficiency, a concern underscored by data from the NDNS Rolling Programme showing that between 2019 and 2023, the only groups within the UK population with evidence of insufficient iodine status were young girls (11‐18 years) and women of childbearing age (16‐49 years) (median concentrations were 95 µg/l and 82 µg/l respectively compared to the World Health Organization's (WHO) criteria for adequate population iodine status of 100–199 μg/L; with 29% and 30% respectively having urinary iodine concentrations <50µg/l versus the WHO target of fewer than 20%) (OHID 2025). Between 2013 and 2023, urinary iodine concentration decreased by 29% for girls (11‐18 years) and 25% for adults (19‐64 years). Iodine is essential for the production of the thyroid hormones thyroxine (T3) and triiodothyronine (T4). Whilst overt deficiency, associated with goitre, growth impairment and mental retardation, is now rarely seen in the United Kingdom, mild to moderate deficiency and insufficiency during pregnancy has been associated with impaired infant neurological development, specifically reduced reading age and IQ in childhood (Bath et al. 2013). In early pregnancy there are significant increases in thyroid hormone production and shifts in iodine usage, leading SACN to state that ‘the iodine status of a woman as she entered pregnancy was arguably as important as introducing iodine‐containing supplements only after the pregnancy had been recognised, and, possibly after a critical period of the fetus' neurodevelopment’ (SACN 2014).

Despite this, the United Kingdom does not currently recommend an increment for iodine intakes either before or during pregnancy, with the original UK Committee of Medical Aspects of Food and Nutrition Policy (COMA) reference value in place since 1991 (140 μg/day) and SACN concluding that there was insufficient evidence, at the point of its review, to support revisions to these dietary reference values (DRVs). Conversely WHO recommends 250 μg/day (WHO 2007) and the European Food Safety Authority (EFSA 2014) recommends 200 μg/day, in both cases with a recommended intake of 150 μg/day for the non‐pregnant adult.

Iodine intakes are affected in part by the iodine content of the soil in which plants are grown and the composition of animal feed used, as well as the use of iodine disinfectants in dairy production. Seasonal as well as geographical variation is commonly seen, making it difficult to accurately estimate the iodine contributed by the diet. Whilst other countries have employed mandatory salt iodisation programmes to address deficiency, access to, and uptake of, iodised salt in the United Kingdom is low and is not formally recommended due to general concerns about salt intake. Instead, the major source of iodine in the UK diet is cows' milk and other dairy products, although white fish and shellfish are also good sources, but intakes are typically lower than dairy products (BDA 2022). Consequently, those who avoid dairy products and/or fish for moral, dietary or religious reasons are at an increased risk of inadequate intake. Plant‐based drinks and dairy alternatives have been increasingly popular in recent years but are inconsistently fortified with iodine, making them an unreliable source and requiring consumer awareness and motivation to choose appropriately fortified options. Seaweed can also be a good source of iodine, but concentrations are hugely variable, with some brown seaweed, such as kelp, containing levels of iodine which could cause excessive intakes (defined as intakes over 600 μg/day (COT 2022a)). As a result, brown seaweed is not recommended to be eaten more than once a week during pregnancy.

Supplementation is not routinely recommended for iodine before or during pregnancy in the United Kingdom, but iodine supplements may be beneficial for anyone who does not regularly consume sufficient iodine‐rich foods. A survey of UK pregnant women living with obesity found that only 42% reported taking an iodine supplement (compared to 56% for vitamin D and 64% for folic acid supplements at the end of the first trimester) (Redfern et al. 2022). Supplementation allowed 100% of this sample to meet the UK recommendations for iodine (and 85.7% to achieve the EFSA guideline) (EFSA 2014) as compared to 15.8% and 14.3%, respectively, of unsupplemented women. Choosing a preconception‐specific multivitamin and mineral supplement (to ensure adequate folic acid (see Section 4.2.2) and appropriate types and levels of vitamin A (see Section 5.5.1)) that contains up to 150 μg/day iodine is advisable, with the remainder coming from the diet, to avoid exceeding upper limits for iodine. Supplemental iodine should be in the form of potassium iodide or potassium iodate and kelp or other seaweed‐based supplements are not recommended.

### 4.3 Key Points

- It is recommended that women who are considering a pregnancy should try to achieve a healthy bodyweight (BMI of 20–25 kg/m^2^) beforehand, as being either underweight or overweight can affect fertility, pregnancy and birth outcomes, with implications for the mother and the fetus/infant.
- Pre‐pregnancy nutritional status is an important determinant of fetal growth and development, and therefore a healthy and varied diet is important for women planning a pregnancy. Certain dietary and food safety recommendations for pregnancy should also be followed prior to pregnancy (see Section 6).
- As it is difficult to get enough vitamin D from food alone during the autumn and winter months, all women in the United Kingdom are advised to consider taking a daily supplement of vitamin D from October to March. High‐risk groups may benefit from supplementation throughout the year.
- It is now well established that taking a folic acid supplement prior to conception and during the first 12 weeks of pregnancy helps prevent the occurrence of NTDs, such as spina bifida.
- Achieving adequate iodine status before conception is important for fetal brain development. Women planning a pregnancy should ensure they include iodine‐rich foods in their diet, including checking any plant‐based dairy alternatives are fortified.

## 5 Nutritional Requirements During Pregnancy

Adequate intakes of energy, protein, vitamins and minerals during pregnancy are required to meet maternal and fetal needs. The developing fetus obtains all its nutrients through the placenta, so dietary intake must meet the needs of the mother as well as the products of conception and enable the mother to lay down stores of nutrients required for the development of the fetus and lactation after birth. However, changes in metabolism during pregnancy lead to more efficient utilisation and absorption of nutrients which means that for many nutrients an increase in dietary intake over and above that which is normally required is not necessary, and pregnant women do not need to ‘eat for two’. For some nutrients, however, an increase in intake is recommended. The UK COMA panel set specific Reference Nutrient Intakes (RNIs) for pregnancy (COMA 1991). Table 4 shows the nutrients for which an increase in requirement has been established by COMA, SACN, NICE or the European Food Safety Authority (EFSA). Pregnant women should be advised on the importance of aiming to meet the recommendations. Those women with low nutrient stores entering pregnancy may be at risk of nutritional deficiencies.

**TABLE 4:** Daily energy and nutrient requirements during pregnancy and nutrients of concern.

	Non‐pregnant women (19–50 years)	Extra requirement for pregnancy	Comments on timing	References
Energy (kcal)	1940	+200	During last trimester	SACN (2011)
Protein (g)	45^a^	+6		COMA (1991)
Omega‐3 fatty acids (EPA + DHA) (mg)	250	+100–200 DHA	From second trimester	EFSA (2010a)
Thiamine (B_1_) (mg)	0.8	+0.1	During last trimester	COMA (1991)
Riboflavin (B_2_) (mg)	1.1	+0.3		COMA (1991)
Folate (μg)	200	+400/100^b^		SACN (2017), NICE (2025)
Vitamin C (mg)	40	+10	During last trimester	COMA (1991)
Vitamin A (μg)	600	+100		COMA (1991)
Iodine (μg)	150	+50		EFSA (2014)
Choline (mg)	400	+80		EFSA (2016)
Vitamin D (μg)	10	+0	Supplement advised from October to March, unless high risk where year‐round supplementation is recommended	SACN (2016), NICE (2025)
Calcium (mg)	700	+0		COMA (1991)
Iron (mg)	14.8	+0		COMA (1991)

Abbreviations: DHA, docosahexaenoic acid; EPA, eicosapentaenoic acid; NTDs, neural tube defects; RNI, Reference Nutrient Intake.

^a^ RNI for adult is 0.75 g/kg/day (45 g/day based on a 60 kg women).

^b^ Pregnant women are advised to take a 400 μg/day supplement of folic acid prior to and until the 12th week of pregnancy (more if at high risk, see Section 4.4.2). The recommendation from 12 weeks onwards is an additional 100 μg.

Healthy eating guidelines for pregnant women are very similar to those for non‐pregnant women with a few exceptions (NHS 2023b). Diets should be based on the UK's healthy eating model, the Eatwell Guide, and include a variety of foods, with plenty of fruit and vegetables, starchy foods particularly wholegrain varieties, sources of protein such as beans, lentils, mycoprotein, nuts, eggs, fish and lean meat, low‐fat dairy products and/or plant‐based alternatives (fortified with calcium and iodine) and a small amount of unsaturated oils. Foods high in saturated fat, salt and free sugars should be limited. Pregnant women should be encouraged to choose foods rich in important vitamins and minerals and a diet with appropriate overall energy intake. It is also important to drink plenty of fluids, preferably water (see Section 5.6).

Low overall adherence to dietary recommendations has been highlighted in all groups of the UK population (Rahmannia et al. 2024), with low intakes of fruit, vegetables, whole grains and fish. In addition, maternal folate, iron and vitamin D intakes in pregnancy are below national nutrient intake recommendations in many regions of the world (Blumfield et al. 2013; Mullaney et al. 2016). Furthermore, women at risk of poorest adherence to dietary guidelines were identified as likely to be smokers, those consuming alcohol and those with elevated BMI (Rahmannia et al. 2024; Blumfield et al. 2013; Mullaney et al. 2016).

**FIGURE 2:**
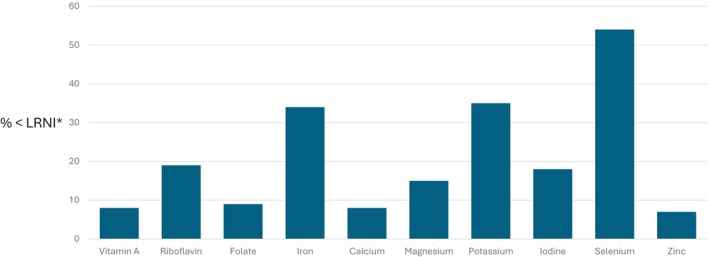
Proportion of women 19‐64 years with nutrient intakes below the lower reference nutrient intake (LRNI).*LRNI: Lower Reference Nutrient Intake ‐ the amount of a nutrient that is enough for only the small number of people with low needs. *Source*: NDNS 2019‐2023 (OHID 2025).

The NDNS, which includes women of childbearing age (non‐pregnant) aged 19–64 years, shows significant proportions of women to have intakes of micronutrients, such as vitamin A, riboflavin, folate, iron, calcium, magnesium, potassium, iodine, selenium and zinc, below the lower reference nutrient intakes (LRNIs) (OHID 2025) (see Figure 2 and Table 8). These data indicate that many women of childbearing age are not meeting their nutrient requirements, and this clearly has implications for pregnancy.

The nutrients of particular concern are those for which there is an increased requirement during pregnancy (protein, vitamin A, thiamine, riboflavin, vitamin C, folate, iodine and choline), based on the recommendations of the report of the COMA Panel on Dietary Reference Values (DRVs) (COMA 1991) and EFSA (2014, 2016), and those where intakes are commonly low (folate, iron and vitamin D). These are discussed in more detail in this section.

### 5.1 Energy Balance and Weight Gain During Pregnancy

In pregnancy, requirements for energy intake are defined as dietary intake necessary to support optimal development of maternal tissues in addition to that required to support fetal growth and development (IOM 2009). Therefore, requirements encompass energy intake that not only balances maternal and fetal energy expenditure but also provides additional energy for fetal growth and development of maternal tissues (Most et al. 2019).

It has been estimated that the total energy cost of pregnancy is approximately 321 MJ (77 000 kcal) across the entire gestational period (FAO 2001). The IOM recommends a total weight gain of 11.3–15.9 kg for those with a healthy weight (see Table 1). SACN suggests that a healthy, well‐nourished woman should gain between 10 and 14 kg during pregnancy (with an average of 12 kg) to increase the probability of delivering a full‐term infant with average birthweight of 3.3 kg and reduce the risk of fetal and maternal complications (SACN 2024). This would indicate that women require a daily increase in energy of approximately 0.35 MJ (85 kcal), 12 MJ (285 kcal) and 2.0 MJ (475 kcal) per day for the first second and third trimesters, respectively (SACN 2011). However, in the United Kingdom, SACN recommendations (SACN 2024) suggest that energy needs do not change in the first 6 months and only increase slightly in the last 3 months and then only by around 200 kcal/day. These recommendations are based on the assumption that fetal growth and GWG stay within the healthy range (SACN 2011). Additionally, energy requirements can vary significantly between individuals and trimesters depending on a range of variables including maternal physical activity levels (Most et al. 2019).

Excessive maternal GWG is associated with several adverse pregnancy outcomes (see Section 2.1), although in the UK women are not routinely weighed during pregnancy unless there is a clinical reason to do so, such as having gestational diabetes. Usual levels of energy consumption for women living with overweight or obesity may already meet or exceed the suggested increments during pregnancy and lactation, and so no further increase in energy may be required (SACN 2024). The NICE Guidelines also concluded that there is not enough evidence to suggest that any particular nutritionally balanced diet is better than another in helping to achieve optimal weight change in pregnancy (NICE 2025).

### 5.2 Protein

Protein is required in pregnancy for fetal growth and cellular development and also for alterations in maternal metabolism and tissue development. Additional protein is needed during pregnancy to support the formation of new blood cells, circulating proteins and maternal tissues and placenta. The total protein requirement during pregnancy has been estimated to be approximately 925 g for a woman gaining 12.5 kg and delivering an infant of 3.3 kg (Hytten 1980). Maternal adaptations occur early in pregnancy involving protein and nitrogen metabolism and evidence suggests that requirements increase, particularly during the second and third trimesters. However, there is a lack of evidence as to exactly how much additional protein is required (Stephens et al. 2015). Thus, protein and amino acid intake recommendations during pregnancy should be gestational stage‐specific, with adequate energy intake to prevent protein from being used as an energy source, ensuring that all nutritional needs are met (Elango and Ball 2016). In the United Kingdom, the DRV for protein intake is increased by an additional 6 g of protein per day throughout pregnancy, bringing the total recommended intake to 51 g per day (COMA 1991). This was based on the Reference Nutrient Intake (RNI) of 0.75 g per kg bodyweight per day for adults, with additional protein requirements during pregnancy. These requirements are based on an average bodyweight of 60 kg which was the average weight of a woman in the United Kingdom at the time (COMA 1991). However, average body weight has risen; for example, the average weight for women in England based on the Health Survey for England in 2022 was 73 kg (NHS England Digital 2024b). Consuming diets high in protein is not recommended due to a lack of data to establish an upper safe intake; therefore, the Department of Health advised that it was prudent for adults to avoid protein intakes which are more than twice the RNI (COMA 1991).

Protein quality is determined by its amino acid composition, bioavailability and digestibility (Mousa et al. 2019). Animal proteins have a higher biological value and are considered ‘complete proteins’ because they provide all nine indispensable amino acids required in the adult diet. In contrast, plant proteins are often referred to as ‘incomplete proteins’ because they typically lack one or more of these essential amino acids in sufficient quantities (Mousa et al. 2019). To ensure a balanced intake of protein, it is recommended to include a range of protein‐rich foods, such as chicken, meat, fish and eggs, as well as a variety of plant‐based options including beans, pulses and lentils (NHS 2022). However, if consuming a variety of plant‐based foods throughout the day, adequate intake of essential amino acids can also be achieved from a plant‐based diet, provided energy intake is sufficient (Mousa et al. 2019). In relation to red meat, current UK guidance concerning the risk of colorectal cancer is to reduce consumption if it exceeds 90 g per day to 70 g or less per day (Public Health England 2018). Evidence linking processed meat to colorectal cancer is stronger than that for unprocessed red meat (WCRF 2018). Processed meats (e.g., sausages, ham and bacon) also tend to have higher amounts of salt and saturated fat, so consumption should be limited (Spiro et al. 2024). Additionally, it is advised to consume two portions (each 140 g cooked weight) of fish per week, including at least one portion of oily fish (limits on fish intake are advised in pregnancy, see Section 6.5).

Although the protein intake of vegetarians and vegans is typically lower compared to omnivores (Wickramasinghe et al. 2024), in the United Kingdom, most women consume more than the recommended DRV of 51 g of protein daily and within the NDNS mean protein intake exceeded the RNI in all age groups of non‐pregnant women (SACN 2024); therefore, it is unlikely that any increase in protein intake is required by most women. A systematic review showed that total protein intake among pregnant women in different countries (the United States and Eastern Mediterranean, Western Pacific, European and South‐East Asian regions), albeit with different assessment instruments of food intake, was 78.21 g/day (95% CI: 74.19–82.44) (Khammarnia et al. 2024).

### 5.3 Fat

During pregnancy, dietary fat plays a crucial role in fetal development, particularly in brain and nervous system formation. The general recommendation is that no more than 35% of total daily energy intake should come from fat, with an emphasis on consumption of unsaturated fatty acids (PHE 2016). Essential fatty acids and their longer‐chain omega‐3 and omega‐6 derivatives are important for fetal growth and development, including brain and eye development (see Section 3.3). Oily fish, such as salmon, mackerel and trout, are rich sources of long chain omega‐3 fatty acids, but the essential omega‐3 fatty acid ALA can be found in foods such as seeds (e.g., flaxseeds and chia seeds), walnuts and walnut oil, soya oil and rapeseed oil. ALA may also be found in smaller quantities in dark green leafy vegetables and in some meats and eggs. Omega‐6 fatty acids, found in vegetable oils (e.g., rapeseed, corn and sunflower), nuts and seeds, support overall cell growth (see Section 3.3 and Table 2). It is also important to limit intake of saturated fatty acids (these should not exceed 11% of daily energy intake), which are commonly found in fatty meats, butter and some cakes, biscuits and savoury snacks. Excessive intakes of these foods can increase the risk of developing obesity and high intakes of saturated fatty acids are a well‐established risk factor for cardiovascular disease.

### 5.4 Carbohydrate and Fibre

Requirements for carbohydrate during pregnancy are not increased and are generally the same as for non‐pregnant women providing approximately 50% of energy intake. Therefore, the Eatwell Guide recommends regular meals based on starchy carbohydrate foods, preferably wholegrain versions (PHE 2016). It is unclear if consuming a low carbohydrate diet (< 175 g/day) safely supports maternal and fetal needs (Sweeting et al. 2021), therefore these diets should be avoided by pregnant women. Pregnant women should aim to eat 30 g/day of dietary fibre as generally recommended for the adult UK population. However, surveys indicate that the majority of women in the United Kingdom do not meet this and, on average, consume 15.5g/day (OHID 2025). Adequate dietary fibre is reported to increase gut microbiome diversity, may aid in reducing excessive GWG and help prevent constipation (Pretorius and Palmer 2020) which is common in pregnancy due to physiological changes (see Section 7.2). High‐fibre foods include those made with whole grains (e.g., wholegrain bread or cereals and whole‐wheat pasta), fruit and vegetables, nuts, seeds and pulses.

Sugars in the diet come from different sources. They can be naturally occurring, for example in fruit and milk, or they can be added in various forms to different foods and drinks. ‘Free sugars’ are those added to foods and drinks by the manufacturer or cook and also those found naturally in unsweetened fruit juices, honey and syrups. The UK government recommends that free sugars make up no more than 5% of daily energy intake, however surveys indicate that many people are consuming around twice as much as the maximum recommended. NDNS data (collected between 2019 and 2023) showed only 17% of adults aged 19‐64 years to be meeting this target. The main sources of free sugars in the UK diet are sugar‐sweetened soft drinks, biscuits, cakes and pastries, desserts, sweets, sweet spreads and confectionery (OHID 2025). Therefore, in general, pregnant women should limit the amounts and portions of these foods/drinks.

### 5.5 Vitamins and Minerals

The DRV panel established increments during pregnancy for vitamins A, C, thiamine (B1), riboflavin (B2) and folate (B9) which are discussed below. No increases were recommended for other vitamins or for minerals, although vitamin D, iron and calcium are thought to warrant extra attention as intakes and status may be low. A recommendation for additional iodine and choline during pregnancy has been made by EFSA (2014, 2016) (see Table 4).

#### 5.5.1 Vitamin A

Vitamin A is a fat‐soluble vitamin found in food as retinol or as vitamin A precursors such as beta‐carotene. Retinol is obtained from animal sources including eggs, dairy foods made from whole milk, oily fish and liver and carotenoids are obtained from plant sources such as dark, leafy vegetables (e.g., spinach) or yellow/orange‐coloured vegetables and fruits including carrots, butternut squash, sweet potatoes, cantaloupe melon and papaya. Beta‐carotene can be converted to vitamin A in the liver, where vitamin A is stored. Extra vitamin A is required during pregnancy for fetal growth and maintenance of eye health and the immune system. The DRV panel concluded that vitamin A intakes should be increased throughout pregnancy by 100 μg/day (to 700 μg/day). This is to allow for adequate maternal storage so that vitamin A is available to the fetus during late pregnancy. However, excess intake of vitamin A can have teratogenic effects on the fetus, increasing the risk of birth defects (see Section 6.1).

The NDNS indicates that some women are not meeting the LRNI for vitamin A (OHID 2025) (see Figure 2 and Table 8). However pregnant women should avoid supplements (including multivitamins) containing retinol (NICE 2025). It is also recommended that pregnant women do not consume liver or liver products, such as liver pate or fish liver oils, as these are rich sources of retinol (Section 6.1) (NHS 2023c).

#### 5.5.2 Thiamine, Riboflavin and Folate

Thiamine (B_1_) and riboflavin (B_2_) are needed for the release of energy in the body's cells. Requirements for thiamine parallel the requirements for energy and are subsequently higher for the last trimester of pregnancy (an increase of 0.1 mg to a total of 0.9 mg/day during the last trimester). The increment for riboflavin requirements is 0.3 mg/day (to a total of 1.4 mg/day) throughout pregnancy. Note that an adaptive response to pregnancy is reduced urinary excretion of some nutrients, including riboflavin, helping to meet the increased demand (see Figure 2 and Table 8 for prevalence of low intakes within the NDNS).

It is recommended that all women who could become pregnant or are already pregnant take folic acid (400 μg) for at least the first 12 weeks of pregnancy (NICE 2025) (see Section 4.2.2). Folate functions as a methyl donor, playing a vital role in DNA synthesis, repair and methylation, and is essential for normal neural tube development and fetal growth. High‐dose folic acid supplements (5 mg a day)^2^ are recommended for those who are at high risk of having a baby with a neural tube defect or other congenital malformation, women who have type 1 or type 2 diabetes or a haematological condition that requires folic acid supplementation, such as sickle cell anaemia or thalassaemia or women taking medicines that can affect the absorption of folic acid, such as anti‐convulsants (NICE 2025). Women who are living with overweight or obesity do not need to take more than 400 μg unless they have the risk factors listed above (NICE 2025). However, those who have had bariatric surgery and are planning a pregnancy are advised to seek specialist advice about folic acid and other micronutrients (NICE 2025).

#### 5.5.3 Choline

Choline is a B vitamin‐like compound that, like folate, functions as a methyl donor and plays a role in cell membrane formation, nervous system development and lipid metabolism (Nguyen et al. 2025). When folate intakes are low, choline requirements increase (NIH 2022). Its importance during pregnancy has drawn attention due to its potential impact on cognitive outcomes and neural tube defect risks (Obeid et al. 2022; Irvine et al. 2022; Mills et al. 2014), though findings are inconsistent. Choline is found in foods such as eggs, meat, fish, poultry, dairy foods, nuts and cruciferous vegetables (e.g., broccoli, Brussels sprouts and cabbage) (Wiedeman et al. 2018).

EFSA recommends an intake of 480 mg/day during pregnancy (EFSA 2016) and in 2023 a health claim was approved for choline's role in liver function for fetuses and breastfed infants (EFSA 2023) and some countries such as the US recommend increased intakes in pregnancy (NIH 2022). However, the United Kingdom does not have a recommended intake for choline as more evidence is needed to establish adequate intake, particularly in the context of pregnancy. Furthermore, choline is not currently included in UK food composition databases and intakes are not reported within the NDNS.

#### 5.5.4 Vitamin C

Vitamin C (ascorbic acid) has important antioxidant functions in the human body. The increase in requirement for vitamin C intake of 10 mg/day (to a total of 50 mg/day) during the last trimester of pregnancy is to ensure that maternal stores are maintained, particularly towards the final stages of pregnancy. The rapidly growing fetus places a moderate extra strain on tissue stores, as it is able to concentrate the vitamin at the expense of circulating vitamin levels and maternal stores. Vitamin C in the maternal diet also has an important role in enhancing the absorption of non‐haem sources of iron (see Section 5.5.7). Pregnant women are therefore encouraged to consume foods or drinks containing vitamin C, together with iron‐containing (non‐haem) meals, in order to help with iron absorption. Vitamin C is found in a wide range of fruits especially citrus and berries and vegetables including tomatoes, peppers and green vegetables.

#### 5.5.5 Vitamin D

The vitamin D status of adult women is maintained more by exposure to sunlight than through diet (NICE 2022a). SACN revised the population recommendations for vitamin D in 2016 (SACN 2016), setting an RNI for all adults of 10 μg/day with no specific additional increment for pre‐conception or pregnancy. Some vitamin D is available naturally in foods, particularly oily fish, but also in eggs and meat. Margarine used to be fortified with vitamin D by law although this requirement ceased in 2013, but it is still voluntarily added to most fat spreads and also some breakfast cereals. There has been an increase in the availability of foods voluntarily fortified or enriched with vitamin D, including bread, eggs and irradiated mushrooms, but the vitamin D content of these is often brand‐specific and the absolute contribution to daily requirements can be relatively low.

Vitamin D is important for the absorption and utilisation of calcium, needed for the calcification of the fetal skeleton, particularly during the later stages of pregnancy and for the prevention of nutritional rickets. More recently a number of non‐skeletal maternal and neonatal outcomes have also been postulated, modulated in part by the role of vitamin D in immunity and potentially established in early pregnancy. The *MAVIDOS* study identified that vitamin D supplementation had a significant positive effect on the neonatal bone mass of winter‐born babies with the effect persisting to 4 years for all supplemented babies irrespective of season of birth (Curtis et al. 2022). However, the evidence for the impact of vitamin D status and/or vitamin D supplementation on outcomes such as pre‐eclampsia, pregnancy‐induced hypertension, birth weight and length remains inconclusive and is still hampered by a limited number of high‐quality trials. Evidence of lower concentrations of circulating vitamin D in neonates relative to their mothers has suggested that the serum levels needed to ensure adequacy may be higher than those applied when modelling requirements in the general population, although an alternative target for optimal health has yet to be defined (Kiely et al. 2020).

Saraf et al. (2016) estimated a global prevalence of vitamin D insufficiency (25(OH)D concentrations < 50 nmol/L) of 54% for pregnant women and 75% for newborns, with almost one‐fifth of pregnant women and newborns collectively being below the 25 nmol/L deficiency cut‐off used in the United Kingdom. In a more recent study of pregnancies in Ireland, 17% of pregnant women and 46% of newborns were below 30 nmol/L, increasing to 49% and 73%, respectively, among ethnic minority groups (Kiely et al. 2016).

Women who receive little sunlight exposure, such as those who cover up their skin or always use high‐factor sunscreen when outdoors, those with darker skin and women living with obesity are at greater risk of having an inadequate vitamin D status, so it is particularly important that they receive supplementary vitamin D during pregnancy. There is evidence of widespread vitamin D deficiency among some minority ethnic groups in the United Kingdom, particularly Black and South Asian infants, contributing to the re‐emergence of nutritional rickets in children from these backgrounds (Buttriss et al. 2021).

In line with the recommendations for the non‐pregnant population, it is recommended that anyone who is pregnant or breastfeeding should take vitamin D (10 μg daily, 400 IU a day) from October to March when sunlight is limited. Those at risk of deficiency may consider taking a supplement throughout pregnancy, including those who have darker skin, and those with little or no exposure to sunshine (NICE 2025).

Vitamin tablets are available to pregnant women for free or at low cost if they are in receipt of NHS Healthy Start payments (see Section 10). Healthy Start vitamin tablets contain folic acid, vitamin C and vitamin D (NICE 2025).

#### 5.5.6 Calcium

Calcium is required for the development of healthy bones and teeth and babies born at full‐term contain approximately 20–30 g of calcium, most of which is laid down during the last trimester. Therefore, fetal growth places increased demands on maternal calcium status, particularly during the latter stages of pregnancy. To meet these demands, physiological adaptations occur, including an increase in maternal free 1,25‐dihydroxyvitamin D3 concentrations (synthesised by the placenta) to enhance calcium absorption, and increased calcium reabsorption in the kidney tubules to enhance calcium retention. Together, these mechanisms enable more efficient uptake and utilisation of calcium. Insufficient calcium intake during pregnancy is reported to increase risks to both fetus and mother.

The UK RNI for calcium for all adults is 700 mg/day, with no increment required during pregnancy. For girls aged 15–18 years, the RNI is 800 mg/day, which accounts for their increased needs for bone growth, but, like adults, no further increase is advised during pregnancy. However, it is essential that pregnant women meet these requirements to ensure that calcium stores and peak bone mass are not compromised. During pregnancy, if calcium intake is insufficient, maternal bone demineralisation can occur, which may be particularly detrimental during adolescence when the skeleton is still developing and increasing in density. Furthermore, any rapid maternal growth spurt may further deplete existing calcium stores. In cases where calcium intake was low during childhood or early adolescence, the body may lack sufficient reserves to meet both the maternal and fetal needs during pregnancy (see Section 7.7).

Emerging evidence suggests that calcium supplements may play a role in reducing the risk of hypertensive disorders during pregnancy, such as preeclampsia and eclampsia, although findings are inconclusive (Wright et al. 2024). Low calcium intake can lead to high blood pressure by stimulating the release of renin and parathyroid hormone. The release of these hormones increases intracellular calcium concentration in smooth muscle cells, causing vasoconstriction, increased peripheral vascular resistance and high blood pressure. Evidence suggests that calcium supplementation is particularly beneficial in women with a low calcium intake who are at a heightened risk of pre‐eclampsia (Perry et al. 2022) which can have severe implications for both the mother and baby (see Section 7.5).

Sources of calcium include milk and dairy products such as cheese and yogurt, green leafy vegetables (e.g., kale, watercress and bok choy), fish containing soft bones (e.g., canned pilchards and sardines), and foods made with fortified flour (e.g., bread). Milk and dairy products are typically good dietary sources of calcium both in terms of content and bioavailability. Alternative dietary sources for vegans and women who do not consume dairy foods include calcium‐fortified plant‐based drinks and yogurt alternatives, almonds, calcium‐fortified soya products and calcium‐set tofu.

#### 5.5.7 Iron

Iron is an essential nutrient required for many functions including the production of haemoglobin in red blood cells, which is necessary for oxygen transport and cellular respiration. Additionally, it is a component of various enzymes involved in energy production and the electron transport chain, DNA and protein synthesis, and has a role in the immune system (Perera et al. 2023). Iron requirements increase during pregnancy to support the development of the placenta and fetus, and the expansion of maternal red blood cell mass (Sangkhae et al. 2023). The UK RNI for iron intake in adult women is 14.8 mg/day, with no additional increment required during pregnancy as this is assumed to be met through cessation of menstrual losses and increased iron absorption. However, pregnant women are at increased risk of iron deficiency and iron deficiency anaemia due to increased blood volume and fetal requirements of pregnancy (see Section 7.3).

Anaemia in pregnancy has been associated with increased maternal and neonatal morbidity and mortality, low birthweight and preterm birth. Anaemia is defined as haemoglobin concentration (Hb) < 110 g/L in first trimester and < 105 g/L in the second and third trimesters (Pavord et al. 2019). Although iron supplementation is commonly recommended to pregnant women in the United Kingdom to prevent iron deficiency anaemia, universal iron supplementation is not recommended for all pregnant women and instead a ‘food first’ approach is favoured, with foods with high iron content being recommended (Pavord et al. 2019).

Data from the NDNS show that 49% of girls aged 11–18 years and 34% of women aged 19–64 have intakes of iron below the LRNI, meaning that many women are likely to enter pregnancy with low iron stores (see Figure 2 and Table 8) (OHID 2025). A recent review of dietary surveys assessing dietary iron intake in pregnant women in Europe revealed that 60%–100% of pregnant women had dietary iron intakes markedly below the recommendation for that country (Milman 2020). Low iron status is reported to be due, in part, to a low and inadequate dietary iron intake (Milman 2020). Many women, including those in the United Kingdom, are reported to enter pregnancy with low iron stores and are at increased risk of iron deficiency during pregnancy (Seymour et al. 2019) with those most at risk including women having successive births, women from lower socio‐economic groups and teenagers. Additionally, a large proportion of pregnant women are reported to have anaemia, with a prevalence of 20%–40% (mean 24.5%) reported in the European region, with iron deficiency anaemia being the predominant cause (Milman 2020).

Pregnant women should be able to get adequate iron by following a healthy diet and consuming foods rich in iron. There are two types of iron in food: haem and non‐haem. Rich sources that contain highly bioavailable haem iron include beef, offal and fish (such as canned sardines and mussels). Sources of non‐haem iron include egg yolks, dark green leafy vegetables, beans, peas, dried fruit, nuts and seeds and some fortified breakfast cereals. However, non‐haem iron is less bioavailable with only around 5% absorbed, depending on the presence of inhibitors (such as phytates, oxalates, calcium and polyphenols) and enhancers in the diet (such as vitamin C and animal protein) (Perera et al. 2023). The UK's Eatwell Guide advises the population to ‘eat less red meat and processed meat’, and those with high intakes (>90g/day) are advised to reduce consumption to no more than 70 g/day due to concerns about risk of colorectal cancer (NHS 2022) (see Section 5.2). Women typically have lower meat intakes compared to men in the United Kingdom and are more likely to adopt plant‐rich diets. This may increase vulnerability to iron deficiency as plant‐based diets contain less haem‐iron and more inhibitors of non‐haem iron absorption (Haider et al. 2018). Those at risk of deficiency should avoid eating foods and drinks, such as tea and coffee and bran which contain polyphenols or phytates that inhibit iron absorption, at the same time as non‐haem iron‐containing foods (see also Section 8.1 on vitamin B12 and plant‐based diets). To optimise the absorption of non‐haem iron from food, it is recommended to consume foods rich in vitamin C, such as citrus fruits, tomatoes and green leafy vegetables at the same meal.

#### 5.5.8 Iodine

Whilst achieving an adequate iodine status prior to pregnancy is recommended (see Section 4.2.3), the same advice applies to pregnancy and women should aim to regularly include ‘pregnancy‐safe’ dietary sources of iodine in their diet such as milk and milk products and fish, particularly white fish (e.g., cod, haddock and coley), as well as other seafood including some shellfish (such as crab and mussels) and oily fish (such as sardines) or suitably iodine fortified plant‐based dairy alternatives (see Section 4.2.3).

### 5.6 Fluid Requirements

Adequate hydration during pregnancy is essential to support the mother's increased physiological demands and the fetus's developmental needs. In the United Kingdom, it is generally recommended that pregnant women consume at least 1.6 L/day (this equates to around eight 200 mL glasses of fluids per day) (NHS Inform 2025), while EFSA recommends 2.3 L/day (which is ≈12 200 mL glasses) (EFSA 2010b). While individual needs can vary depending on factors such as physical activity, climate and overall health, staying hydrated is essential to support increased blood volume, amniotic fluid production, and the removal of waste products, as well as to help reduce the risk of common pregnancy‐related issues such as constipation and urinary tract infections (see Section 7.2).

Pregnant women should be encouraged to drink fluids regularly throughout the day, with water being one of the best choices, though other fluids such as milk can be useful to supply important nutrients (see above). Caffeine should be limited, and it is recommended to consume a maximum of 200 mg/day (see Section 6.3). Some of the herbs used in herbal teas should not be consumed in high amounts, especially during the first trimester of pregnancy. The NHS recommends that, as a general rule, up to 1 to 2 cups of herbal and green tea a day during pregnancy is safe to consume (NHS 2023c) (see Section 6.7).

Frequent consumption of sugar‐sweetened beverages can significantly increase calorie intake and may contribute to excess energy consumption. Pregnant women are therefore advised to limit intakes of fizzy drinks or other drinks that are high in sugar. Drinks containing approved low‐calorie sweeteners are considered safe for consumption during pregnancy when consumed within the acceptable daily intake levels (EFSA 2023). However, pregnant women should prioritise water, milk and no‐added‐sugar fruit juice (limited to 150 mL/day) to support both hydration and nutritional needs.

### 5.7 Key Points

- To ensure optimal maternal and fetal health, adequate intake of energy, protein, essential fatty acids, vitamins and minerals during pregnancy is essential, as the fetus relies entirely on the mother's nutrient supply.
- While metabolic changes in pregnancy enhance nutrient absorption, certain nutrients such as energy, protein and essential fatty acids, as well as vitamins A, C, thiamine, riboflavin, folate, choline and the mineral iodine require increased intakes and others such as iron, calcium and vitamin D are thought to warrant extra attention as intakes and status may be low.
- UK guidelines suggest an increase of around 200 kcal/day in the third trimester to support fetal growth and maternal tissue development. Pregnant women do not need to ‘eat for two’ and do not need a special diet in pregnancy (NICE 2025), although a more nutrient‐dense diet may be needed to meet nutritional requirements. Usual levels of energy consumption for women living with overweight or obesity may already meet or exceed the suggested increments during pregnancy and lactation and so no further increases are required. However, weight loss is not recommended during pregnancy.
- Protein needs increase during pregnancy, and an emphasis should be placed on high‐quality animal proteins and varied plant‐based sources. Additionally, the long‐chain omega‐3 fatty acids found in oily fish are important for fetal brain and eye development. Carbohydrate and fibre requirements remain the same as for non‐pregnant women, with a recommended 30 g of fibre per day to support digestive health and prevent complications such as constipation during pregnancy.
- Certain vitamins and minerals require careful attention. Intake of vitamin A, thiamine, riboflavin, folate and vitamin C should increase slightly to support fetal growth and energy metabolism, though excessive vitamin A intake should be avoided (see Section 6.1). Folic acid supplements are recommended preconception and for the first trimester to prevent neural tube defects. Although no additional iodine intake is officially recommended in the United Kingdom, EFSA advises an increase due to its importance in fetal brain and nervous system development. EFSA also recommends an increase in choline intakes, although this is not currently advised in the United Kingdom.
- Vitamin D is critical for calcium absorption and fetal bone development. In the United Kingdom, it is recommended that all pregnant women consider taking a daily supplement of 10 μg, whilst year‐round supplementation may be advised for those at higher risk (NICE 2025).
- While no additional increases are suggested for most other vitamins and minerals, calcium and iron deserve special attention. This is because they play vital roles in fetal development and maternal health, and inadequacies are relatively common during pregnancy.
- Pregnant women should follow healthy eating guidelines similar to those for non‐pregnant women, with a focus on nutrient‐dense foods. The UK's Eatwell Guide emphasises starchy foods particularly those made from whole grains, fruits, vegetables, lean protein‐rich foods and lower‐fat dairy products, while minimising foods high in fat, salt and sugar. Diet quality, variety and balanced energy intake are key to meeting nutrient needs.
- Despite these guidelines, data from the NDNS shows that many women of childbearing age fail to meet recommended intake levels for key micronutrients, which can negatively affect pregnancy outcomes. Common inadequacies include iron, folate, calcium and iodine, often exacerbated by poor dietary habits and risk factors such as smoking, alcohol consumption and elevated BMI.
- Fluid intake is also crucial during pregnancy. In the United Kingdom, pregnant women are generally advised to consume at least 1.6 L (this equates to around 8 glasses) of fluids per day to support both maternal and fetal health.

## 6 Food Safety Issues During Pregnancy

Pregnant women are advised to pay particular attention to food hygiene during pregnancy and to avoid certain foods in order to reduce the risk of exposure to substances that may be harmful to the developing fetus. Potentially harmful substances include foodborne pathogens (e.g., *Listeria monocytogenes* and Salmonella) and toxic food components (e.g., dioxins and polychlorinated biphenyls [PCBs]), as well as alcohol and high doses of some dietary supplements (e.g., vitamin A).

### 6.1 Vitamin A

Excessive intakes of vitamin A in the form of retinol are a concern during pregnancy, principally in developed countries, due to possible teratogenic effects (COT 2022b). The main effects associated with excessive vitamin A intake, particularly during early pregnancy, are congenital malformations involving the central nervous and cardiovascular systems and spontaneous abortion (Maia et al. 2019), although findings of human studies remain mixed (COT 2022).

EFSA set a tolerable upper intake level (UL) for vitamin A of 3000 μg/day, which applies to women of childbearing age including those who are pregnant or lactating, based on the risk of teratogenicity and liver damage (EFSA 2006, 2015). The UK Expert Committee on Vitamins and Minerals (EVM) did not recommend a maximum level of intake but, considered an intake greater than 1500 μg/day to be ‘inappropriate’, based on possible effects on bone for the population as a whole (COT 2003).

The most common way that pregnant women and those considering pregnancy might be exposed to high dietary doses of vitamin A is from food supplements (vitamin pills and fish liver oil supplements). At a global level, WHO recommends that vitamin A supplements should not be given to pregnant women except to prevent night blindness in places where vitamin A deficiency is a severe public health problem (which does not include the United Kingdom) (WHO 2016).

Some foods, namely liver and liver products, can also have a high vitamin A content. A typical 100 g portion of fried calves' liver contains 25 217 μg of retinol, whilst the same weight of fried chicken liver contains 10 500 μg (FSA 2021). This is thought to be due to retinol being added to animal foodstuffs to aid productivity, reproduction and immune status. No other food provides as much vitamin A, although some fortified spreads and other fortified health foods may, in combination, provide more than the recommended limit (COT 2022b).

The UK Government therefore recommends that, in order to avoid possible harm to the unborn child, pregnant women or women thinking about having a baby or trying to conceive, should avoid consuming liver or liver products such as paté or liver sausage or taking supplements that contain retinol (including multivitamins and fish liver oils).

It is noteworthy that most supplements contain beta‐carotene instead of retinol and excess intake of beta‐carotene does not lead to increased plasma retinol concentrations because of its low conversion rate. A high intake of beta‐carotene has not been found to be associated with congenital defects (Miller et al. 1998; COT 2022b).

A healthy and varied diet should provide sufficient vitamin A in the UK population. Sources of vitamin A in the diet apart from liver and liver products (e.g., dairy products made with whole milk, eggs, leafy vegetables and orange‐coloured fruit and vegetables such as carrots and melon) do not pose any risk of excessive intakes, and should be included as part of a healthy, balanced diet during pregnancy.

### 6.2 Alcohol

Alcohol has negative impacts on a number of reproductive processes, from early oocyte and sperm development, through to pregnancy viability and duration, fetal growth and infant and future child health. The UK has one of the highest rates of alcohol drinking during pregnancy at between 41.3% and 75% (Gomez et al. 2022). It also has one of the highest rates of fetal alcohol spectrum disorder (FASD, 3.2%), resulting from prenatal exposure of the developing brain and body to excessive alcohol (Schölin et al. 2021). FASD has more than 400 potential co‐morbid conditions with lifelong implications and, whilst there are characteristic facial features, these may only affect 10% of sufferers meaning diagnosis is often delayed. FASD leads to restricted growth and learning and behavioural disorders but, even at lower levels of alcohol exposure, there is increased risk of miscarriage, still birth, premature birth, small for gestational age and low birthweight. Although attention has commonly focused on the risks associated with high intakes and binge drinking, low but frequent alcohol consumers represent a large but potentially under recognised ‘at risk’ population (Department of Health 2016a; Royal College of Obstetrics and Gynaecologists 2015). UK studies report that over half of women were consuming more than 2 units of alcohol per week in their first trimester and one quarter drank alcohol after confirmation of their pregnancy (Nykjaer et al. 2014; Smith et al. 2014). Whilst high risk drinking behaviour and specifically binge drinking are consistently associated with a ‘risk phenotype’ that may include co‐occurring illicit drug use, smoking, low income and/or education, low age, previous stressful life events and being unmarried (Mullally et al. 2011; Naimi et al. 2003; Witt et al. 2015; Iversen et al. 2015), any drinking may be more common in more educated women and/or those classified as higher socio‐economic or lower deprivation status (McCormack et al. 2018; Symon et al. 2016). Older mothers (35–39 and 40 years or over) were also more likely to consume alcohol during pregnancy (PHE 2020).

The UK Chief Medical Officer released revised guidance in 2016 advising pregnant women and those planning pregnancy to abstain from alcohol, citing a lack of evidence to support a safe amount or safe timing of drinking during pregnancy, combined with no known benefits of alcohol consumption (Department of Health 2016b). Whilst this brings UK advice in line with that of other countries, such as Australia and Canada, it was at odds with previous advice and was not adopted by NICE for 3 years, leading to uncertainty among health professionals and the general public and a general lower priority afforded to reducing alcohol consumption than to other behaviour modifications such as smoking cessation.

Standards published in 2022 aiming to improve the assessment and diagnosis of FASD and to improve support during pregnancy to prevent FASD encourage health professionals to advise women throughout pregnancy not to drink alcohol and to ask them about their alcohol use throughout their pregnancy and ensure these conversations are recorded (NICE 2022b).

Unfortunately, beyond screening, there are limited interventions proven to reduce alcohol consumption in those planning a pregnancy or already pregnant, although recognised behaviour change approaches, such as tailored interventions and motivational interviewing, are recommended.

Indirect or exacerbated effects of alcohol consumption may also be caused by co‐occurring micronutrient deficiencies. Requirements for B vitamins are increased yet intakes may be low in heavy drinkers, while serum antioxidant levels can be reduced, limiting the protection provided against free radicals in maternal and fetal tissues. Of particular note is the impact of alcohol on the absorption and utilisation of folate, compounding inadequate peri‐conceptual folate status.

### 6.3 Caffeine

Caffeine is a psychoactive alkaloid that freely crosses the placental barrier. Although caffeine is metabolised in the liver, the fetal liver and placenta lack the necessary enzymes to break it down, resulting in prolonged fetal exposure. While there is inter‐individual variability, pregnancy is associated with an increased half‐life of caffeine, reaching 9–11 h in the third trimester (Grosso and Bracken 2005). This extended half‐life raises concerns about potential adverse effects, particularly in relation to suggested links with spontaneous abortion and low birthweight (Dube et al. 2025), although the evidence base remains inconclusive and a causal relationship has not been definitively established (Brito Nunes et al. 2023).

Current evidence does not support an association between caffeine intake and prematurity. While caffeine intake has been linked in some studies to impaired fertility, miscarriage and stillbirth, the risk attributable to caffeine appears to be lower and/or the evidence base weaker compared to other established risk factors (e.g., for miscarriage: advanced maternal age [> 30 years], extremes of body weight; for stillbirth: maternal obesity, smoking and undetected fetal growth restriction).

Current UK guidance, reviewed by the Royal College of Midwives (RCM) in 2022, states that pregnant women can safely consume up to 200 mg of caffeine daily (RCM 2022). Up until 2008, the recommended maximum intake was 300 mg/day, but this was reduced following review by the Committee on Toxicology (COT) on the basis of further evidence linking caffeine intakes above 200 mg/day with fetal growth restriction (COT 2008). However, as with alcohol, it is not possible to identify a threshold below which there is no elevation of risk and for caffeine specifically this is made more difficult by the limited and heterogeneous studies available (in methodology and outcomes), which often lack standardisation as to what and how intakes are assessed or assumed. There is substantial intra‐ and inter‐individual variation in the effects of caffeine due to a range of factors including metabolism and therefore rate of clearance from the body, smoking status and accompanying food intake and also the potential for any ‘caffeine effect’ to in fact be mediated by other components of the food or drinks or other associated lifestyle behaviours, such as alcohol consumption and bodyweight, which may not be adequately controlled for in studies.

In practical terms, women planning a pregnancy or already pregnant should be encouraged to reduce their caffeine intake to 200 mg/day or less and provided with practical tools, such as online caffeine calculators to facilitate this. All food and non‐food sources of caffeine should be considered (see Table 5), as well as the potential for caffeine contents to vary substantially even within one category (e.g., coffee), due to brewing method, brand and strength. Suitable low or no caffeine alternatives should be suggested and health professionals should be aware that, for some women, coffee may not be the primary caffeine source in the diet. Energy drinks can contain higher levels of caffeine than other soft drinks and are not recommended during pregnancy. Caffeine is also found in a number of prescription and over‐the‐counter medicines (e.g., headache pills, cold and flu remedies, diuretics and stimulants) and women are advised to check with their GP or other health professional before using such medicines.

**TABLE 5:** Estimated caffeine contents of UK and European food, drinks^a^ and medicines. Caffeine content and portion sizes vary within and between countries, but the following amounts serve as useful guidelines.

Product	Common variations	Typical caffeine content mg per 100 mL/100 g (made up)	Typical caffeine content per portion [estimated portion size]	No or lower caffeine alternatives
Hot drinks^a^				
Coffee	Instant Espresso Brewed/Filter	45 133 45–56	113 mg [250 mL] 80 mg [60 mL] 113–140 mg [250 mL]	Decaffeinated coffee
Tea	Black	22–30	55–75 mg [250 mL]	Decaffeinated tea
Herbal tea	Green tea (bags/leaves) Matcha tea powder	13–18 33–78	30–40 mg [227 mL] 76–178 mg [227 mL]	Decaffeinated green tea
Hot chocolate	Instant (made with water) Made with cocoa powder	0–2 ≤ 10	0–5 mg [260 mL] ≤ 25 mg [260 mL]	Instant hot chocolate powders
Cold drinks				
Energy drinks		32	80 mg [250 mL]	
Cola		11.2	28 mg [250 mL]	Caffeine free cola
Foods				
Milk chocolate		20	10 mg [50 g]	
Plain chocolate		50	25 mg [50 g]	
Over the counter medicines that may contain caffeine^b^				
Cold and flu remedies^c^	Capsules Drink powders	N/a	25 mg/capsule 50 mg/sachet	
Painkillers^c^	For example, Anadin Extra, Alka‐Seltzer	N/a	15–65 mg/capsule	
Stimulants	For example, ProPlus	N/a	50 mg/tablet	

^a^ Caffeine in drinks will be particularly variable as affected by factors such as brand, brewing method and time.

^b^ Caffeine must be listed in the ‘active ingredients’ section of product labels. Products may include advisory statements such as ‘Contains caffeine. Not recommended for children or pregnant women’, especially if the caffeine content is significant.

^c^ ‘Extra’ in names of products such as painkillers and old and flu remedies often means higher caffeine contents.

*Source*: EFSA (2025), Datapharm (2025), Čížková et al. (2008), Koláčková et al. (2020), NHS (2023c), FSA (2024).

### 6.4 Foodborne Illness

The following food pathogens can cause potential harm to the developing fetus during pregnancy. This information is also summarised in Table 6.

**TABLE 6:** Summary of foodborne pathogens that can cause potential harm in pregnancy.

Consequences of infections in pregnancy	Foods to avoid	Further comments
**Listeriosis** Can cause miscarriage, stillbirth or severe illness in the newborn	Pâté and mould‐ripened soft cheeses (e.g., Brie, Camembert, chevre), blue‐veined cheeses (e.g., Danish Blue, Gorgonzola) (unless steamed until boiling hot before eating); unpasteurized milk and milk products; cold cured meats (such as salami, pepperoni, chorizo and prosciutto) and smoked cured fish unless these have been cooked thoroughly (pre‐packed cold meat such as ham is safe); sushi, unless the fish has been thoroughly cooked or frozen	Destroyed by heat, so re‐heat ready‐prepared meals thoroughly. Wash fruit and vegetables well
**Salmonella** In severe cases may cause miscarriage or premature labour	Raw eggs or foods containing raw or partially cooked eggs, for example, home‐made mayonnaise (unless British Lion eggs)	Choose eggs with a British Lion logo stamp. Cook all other eggs until white and yolk are solid. Cook meat, particularly poultry, thoroughly
**Toxoplasmosis** In rare cases can lead to severe fetal abnormalities	Raw or undercooked meat; liver and liver products, pâté, game meats, cold cured meats (such as salami, pepperoni, chorizo and prosciutto) (unless cooked) and unpasteurised (‘raw’) milk and milk products	Avoid contact with soil or cat litter trays by wearing gloves
**Campylobacter** May cause premature birth, spontaneous abortion or stillbirth	Raw or undercooked poultry; unpasteurised milk and milk products	Domestic pets and soil can also be a source of infection

#### 6.4.1 Listeriosis

Listeriosis is a flu‐like illness caused by the bacteria *Listeria monocytogenes* . Although listeriosis is rare in the United Kingdom, if it occurs during pregnancy, it can cause miscarriage, stillbirth or severe illness in the newborn. Pregnant women are therefore advised to avoid foods in which high levels of the bacteria have sometimes been found, most of which are chilled, ready‐to‐eat foods. This includes pâté and soft cheeses which are mould‐ripened (e.g., brie, camembert or chèvre (a type of goat's cheese)) and soft blue‐veined cheeses (e.g., Danish blue, gorgonzola, roquefort), unless cooked until steaming hot before eating. Hard cheeses such as cheddar and other cheeses made from pasteurised milk pose no risk, including cottage cheese, mozzarella and processed cheese spreads. Other higher risk foods for Listeria infections include cold, cooked sliced meats and cured meats (unless cooked thoroughly), smoked and cured fish (including sushi) unless the fish has been thoroughly cooked and pre‐cooked and chilled shellfish. Unpasteurised milk and milk products (such as soft‐ripened goats' cheese) should also be avoided.

Listeria bacteria are destroyed by heat and therefore, as with the general population, pregnant women are advised to reheat ready‐prepared meals thoroughly, particularly if they contain poultry. Cooking cheese until it is steaming hot will reduce the risk of listeriosis. Ready‐prepared foods which will not be reheated can also be higher risk (e.g., purchased salads, pre‐cut fruit and pre‐prepared sandwiches). It is also advisable to wash fruit and vegetables very thoroughly, especially if they are to be eaten raw, in order to minimise risk.

#### 6.4.2 Salmonella

Salmonella is a major cause of food poisoning in the United Kingdom (Holland and Mahmoudzadeh 2020), and in severe cases, it may cause miscarriage or premature labour in pregnant women. Salmonella poisoning is most likely to come from raw eggs or undercooked poultry; therefore, pregnant women are advised to avoid eating raw eggs without the British Lion logo stamp or foods containing raw or partially cooked eggs (e.g., homemade mayonnaise and some cold desserts). Eggs with the British Lion logo stamped on their shell are considered very low risk for Salmonella and safe for pregnant women to eat raw or partially cooked (NHS 2023c). However, all other eggs should be cooked until the white and yolk are solid. All meat, in particular poultry, needs to be thoroughly cooked, and pregnant women should take particular care when handling these foods. Raw foods should be stored separately from cooked foods in the fridge (so that raw foods cannot drip on cooked foods) in order to avoid the risk of cross‐contamination.

#### 6.4.3 Toxoplasmosis

Toxoplasmosis is a condition caused by the organism *Toxoplasma gongii* which can be found in raw meat, unpasteurised milk and cat faeces. Infection in pregnancy can, in rare cases, lead to severe abnormalities in the fetus, including blindness and mental retardation. Pregnant women are advised to avoid unpasteurised milk and milk products (particularly goat's milk), raw or undercooked meat, liver and liver products, pâté (including vegetarian varieties) and game meats such as goose, partridge or pheasant as a precaution. They should also be careful with cold‐cured meats, such as salami, pepperoni, chorizo and prosciutto, unless these are cooked thoroughly.

In addition, they should avoid contact with soil or cat litter trays by wearing gloves when gardening or changing cat litter. Pregnant women should be particularly cautious about food hygiene if cats come into their kitchen.

#### 6.4.4 Campylobacter

Campylobacter is a genus of pathogenic organisms which are a common cause of food poisoning in the United Kingdom. Infections during pregnancy have been associated with premature birth, spontaneous abortion and stillbirths. Common sources of infection include poultry, unpasteurised milk, untreated water, domestic pets and soil. As with other sources of infection, observing good hygiene practices helps to reduce risk.

### 6.5 Fish

Whilst there is a general recommendation for the UK population to eat at least two portions of fish a week, one of which should be oily fish, since 2004 there has been specific advice around maximum intakes and type of fish for certain groups including pregnant and breastfeeding women (SACN 2004).

The basis for the recommendations on fish for the general population is that the consumption of fish, particularly oily fish, confers significant health benefits, in terms of a reduction in the risk of cardiovascular disease. This is thought to be due, at least in part, to the long‐chain *n‐*3 PUFAs found in fish, levels of which are particularly high in oily fish (SACN 2004). In addition, these long‐chain fatty acids are required for the development of the central nervous system in the fetus and young infant, so are important for both pregnant and breastfeeding women (see Section 3.3). However, a maximum limit on oily fish consumption is advised due to the risk of exposure to pollutants, such as dioxins and PCBs, which have been found in oily fish. These are persistent compounds that may accumulate over time in the body and could have adverse health effects if consumed at high levels over a long period of time. Pregnant and breastfeeding women (and women who may become pregnant) should therefore limit their intake to no more than two portions of oily fish per week. A portion is around 140 g cooked weight (NHS 2023c).

Pregnant women, and those who may become pregnant, are also advised to avoid marlin, shark and swordfish due to the risk of exposure to mercury, which at high levels can be harmful to the developing nervous system of the fetus (Chen and Dong 2022). It is recommended that other adults, including breastfeeding women, restrict intake of these fish to no more than once per week. Mercury, in the form of methylmercury, is concentrated up the food chain and therefore found in the highest amounts in large, predatory fish. The highest concentrations have been found in shark, marlin and swordfish but tuna also contains higher levels of mercury than other fish and the FSA advises pregnant women to limit their intake to no more than two portions of fresh tuna per week (a portion is 140 g cooked steak) *or* four medium‐sized tins of tuna per week (each with a drained weight of around 140 g). There are no restrictions advised for tuna for breastfeeding women. Pregnant women may also want to moderate their intake of other fish that may contain higher amounts of mercury such as sea bream, sea bass, turbot, halibut and rock salmon (also known as dogfish, flake, huss, rigg or rock eel).

### 6.6 Food Allergy

Peanut allergy receives widespread attention because it is the most common cause of severe allergic reactions to foods. As many as 2% or 1 in 55 children in the United Kingdom show evidence of an allergic reaction to peanuts (Miles and Buttriss 2010; Grabenhenrich et al. 2020). Unlike many food allergies, which tend not to persist beyond childhood, peanut allergy is often a life‐long problem and can cause severe anaphylactic reactions to tiny amounts of peanut protein. The cause of peanut allergy is still unclear, but infants with eczema and/or egg allergy appear to be more at risk. Those whose parents have a history of allergic disease are more likely to develop allergies themselves. True IgE‐mediated allergic reactions such as peanut allergy require prior exposure and sensitisation of the immune system to the allergen. Since peanut‐allergic infants tend to show symptoms on their first known exposure to peanut, it has been suggested that sensitisation to peanut may be acquired by the fetus during pregnancy, via foreign proteins crossing the placenta (or via breastmilk during lactation) or by an unrecognised dietary exposure or non‐oral (skin or respiratory) routes (e.g., via eczematous skin). However, it has also been postulated that exposure to foreign proteins via the placenta may be important in helping the baby to develop a tolerance to the many foreign proteins in the environment.

Advice used to be that if there is a strong family history of atopic disease (i.e., if either parent or a previous child has suffered from hay fever, asthma, eczema or other allergy), then it may be advisable to avoid peanuts or foods containing peanuts during pregnancy and while breastfeeding, in order to reduce the risk of the infant developing a peanut allergy (FSA 2002). However, since then, reviews of studies investigating this association suggest that there is no clear evidence that eating or not eating peanuts (or foods containing peanuts) or non‐dietary exposure to peanuts during pregnancy or breastfeeding has any effect on the chances of a child developing a peanut allergy (Thompson et al. 2010; Halken et al. 2021). Similarly, most trials have found no reduction in the prevalence of food allergy in infants of women avoiding other dietary allergens such as egg and milk (Halken et al. 2021).

Current advice is that pregnant and breastfeeding women can consume foods that can trigger allergic reactions, including peanuts, if they wish to do so unless they are allergic to them themselves or have been advised to avoid them by a health professional. This includes peanuts and peanut products (such as peanut butter), which can be included during pregnancy as part of a healthy, balanced diet. Any diet that excludes specific foods or food groups may be restrictive in terms of nutrient intake and should only be followed under medical supervision.

### 6.7 Herbal Medicines

Herbal medicinal products, which are any plant‐derived product (i.e., leaves, roots and flowers), should be consumed with caution during pregnancy (Muñoz Balbontín et al. 2019). Pregnant women are advised to avoid the herbal remedy liquorice root (NHS 2023c), as it has a particularly high concentration of glycyrrhizin; some studies suggest that high intakes can increase the risk of miscarriage, early delivery and stillbirth (Nazari et al. 2017).

### 6.8 Key Points

- Excessive intakes of vitamin A, in the form of retinol, are toxic to the developing fetus and may cause birth defects; therefore, pregnant women or those who may become pregnant, are advised to limit their intake of vitamin A by avoiding liver and liver products and supplements containing retinol unless specifically recommended by a healthcare provider. Vitamin A from plant‐based sources (beta‐carotene), found in some fruits and vegetables is not associated with the same risks as retinol.
- All women of childbearing age and all professionals who deliver their healthcare and education should be aware that the only safe option regarding alcohol is total abstinence from before conception and throughout pregnancy.
- Whilst binge drinkers and those with alcohol dependency are specific at‐risk groups, any alcohol consumption in the peri‐conception period can affect fertility and will pose a risk to the health of any subsequent fetus.
- Intakes of caffeine should not exceed 200 mg/day. There are many sources of caffeine in the UK diet, and the composition of foods, drinks and medicines can vary substantially.
- Pregnant women are advised to pay particular attention to food hygiene and to avoid certain foods during pregnancy in order to minimise the risk of food poisoning from potentially harmful pathogens, such as *L. monocytogenes* and Salmonella.
- Pregnant women and those who may become pregnant are advised to consume no more than two portions of oily fish per week, to avoid exposure to dioxins and PCBs. They should also avoid shark, marlin and swordfish and limit their intake of tuna to prevent exposure to methylmercury, which can be harmful to the developing fetus.
- Pregnant women with known food allergies should continue to avoid those allergens during pregnancy to prevent allergic reactions. The advice regarding allergen avoidance during pregnancy, particularly concerning peanuts, has evolved over time. Current guidance is that there is no need for pregnant women to avoid peanuts or other allergens unless they themselves have a known allergy to that food.
- Herbal medicinal products should be consumed with caution during pregnancy and pregnant women are advised to avoid the herbal remedy liquorice root, as it has a particularly high concentration of glycyrrhizin.

## 7 Diet‐Related Conditions During Pregnancy

### 7.1 Nausea and Vomiting and Changes in Taste and Appetite

Nausea and vomiting are common symptoms experienced during pregnancy, particularly during the first trimester and are reported to occur in 50%–90% of women. Nausea and Vomiting of Pregnancy (NVP) is defined as the symptom of nausea and/or vomiting during pregnancy when the onset is prior to 16 weeks of gestation and where there are no other causes. Hyperemesis Gravidarum (HG) is a severe form of NVP, which affects between 0.3% and 3.6% of pregnant women, interfering with quality of life and the ability to eat and drink normally (Nelson‐Piercy et al. 2024). The causes of these symptoms are unknown, although recent evidence suggests that hormones, including GDF15, which is produced by the placenta and causes a loss of appetite and nausea, are responsible (Fejzo et al. 2024). Some women experience worse symptoms due to genetic differences, which cause them to have higher levels of, or greater sensitivity to, GDF15 (Nelson‐Piercy et al. 2024; Fejzo et al. 2024). It is advised that women with NVP but who are not dehydrated eat little and often to prevent an empty stomach, eat small, frequent, high‐carbohydrate meals and snacks (such as sandwiches and breakfast cereal) and fluids such as milk between meals and avoid foods and smells that precipitate the symptoms (British Dietetic Association 2019).

Changes in taste and appetite are also common in pregnancy. Cravings and aversions to foods are frequently reported, with the most common cravings being for sweet foods, especially chocolate, fruit and fruit juices and dairy foods (Hill et al. 2016). However, such cravings do not seem to impact overall dietary and nutritional intake (Hill et al. 2016). Common aversions include tea and coffee and alcohol (British Dietetic Association 2019). Pica is the craving and consumption of non‐food substances, such as soap, coal and chalk, and the reasons for the development of this are unknown. Pica is more common in low‐ and middle‐income countries compared to high‐income countries (British Dietetic Association 2019).

### 7.2 Constipation

Constipation is commonly reported in pregnant women. The causes are complex and likely to be due to physiological effects on gastrointestinal function caused by pregnancy. Changes in hormone levels, particularly increased progesterone, are responsible for reduced intestinal smooth muscle motility (Verghese et al. 2015) and increased gut transit time (British Dietetic Association 2019). Pregnant women are advised to increase their intake of fibre‐rich foods, in particular, whole grain foods, pulses, fruit and vegetables and aim to eat the recommended 30 g fibre/day (SACN 2015). Women should drink plenty of fluids and take gentle exercise in order to alleviate the condition. Other considerations include the use of dietary bulking agents and, if iron supplements have been recommended, changing type may also be beneficial as iron supplements may sometimes aggravate the symptoms of constipation (British Dietetic Association 2019).

### 7.3 Anaemia

Anaemia is defined as a low haemoglobin concentration. In pregnancy, there is a physiological expansion of plasma volume which exceeds the increased production of red blood cells and haemoglobin (see Section 2.2). The resulting haemodilution contributes to the fall in haemoglobin during pregnancy. Anaemia in pregnancy can be caused by numerous other factors, including vitamin B12 and folate deficiency, thalassaemia, blood loss, and, most commonly, a deficiency of iron. In the United Kingdom, anaemia in pregnancy is defined as haemoglobin < 110 g/L (Pavord et al. 2019). Recent national audit data indicate that iron deficiency anaemia affects over 30% of pregnant women in the United Kingdom, highlighting a persistent public health issue (Churchill et al. 2022). Maternal anaemia has been associated with a significantly higher risk of perinatal and neonatal mortality, low birthweight and preterm birth (Pavord et al. 2019). A healthy diet with iron‐rich foods may help prevent iron deficiency anaemia during pregnancy. Dietary sources of haem iron include red meat and fish which is absorbed two to three times more readily than non‐haem iron sources which include eggs, dark green leafy vegetables, pulses, nuts and seeds and iron‐fortified breakfast cereals. Vitamin C (ascorbic acid) significantly enhances the absorption of non‐haem iron and is found in foods such as fruit, especially citrus fruit and vegetables for example broccoli, cabbage and peppers. However, tannins in tea and coffee inhibit iron absorption when consumed with a meal or shortly after. In practice, many women are prescribed iron supplements by medical professionals at some point during pregnancy. However, they may cause side effects such as nausea and constipation and their use requires monitoring (see Sections 7.1 and 7.2).

### 7.4 Gestational Diabetes

Gestational diabetes mellitus (GDM) is one of the most common complications in pregnancy and is recognised as a global health problem. GDM is defined as any degree of glucose intolerance with onset or first recognition during pregnancy (WHO 2013). Many countries have observed a rapid increase in the prevalence rates, with global prevalence reported as 14% (Plows et al. 2018; Wang et al. 2022); however, prevalence rates vary due to variation in screening strategies and diagnostic criteria (Wang et al. 2022).

GDM can develop at any time during pregnancy, although it is more frequently diagnosed in the second and third trimesters and is usually diagnosed following an oral glucose tolerance test. GDM is associated with a higher incidence of adverse maternal and fetal outcomes, including obstetric complications and difficulties during labour, perinatal and neonatal morbidities including macrosomia (birthweight > 4.5 kg) and a 50% increased risk of subsequent development of type 2 diabetes in the mother (Vounzoulaki et al. 2020). Women living with obesity (BMI > 30 kg/m^2^) are at increased risk of developing GDM, as are those from ethnicities with a high prevalence of diabetes, those with previous gestational diabetes or those who have previously had a macrosomic baby (NICE 2020).

If GDM is diagnosed during pregnancy, treatment is aimed at achieving appropriate blood glucose levels, while conducting regular fetal and obstetric monitoring to reduce the risk of maternal and fetal complications (NICE 2020). Dietary management focuses on consuming a healthy diet for pregnancy (see Section 5), with a specific focus on the type, amount and distribution of carbohydrate during the day to reduce glycaemic load and subsequently, postprandial blood glucose levels (Dyson et al. 2018), although there is a lack of consensus regarding a specific diet for GDM management (NICE 2025). Therefore, it is recommended to regularly consume low glycaemic index carbohydrates, which are usually higher in fibre, while restricting intake of foods high in sugar (Dyson et al. 2018). As many women with GDM are living with overweight or obesity, it is also important to manage weight gain during pregnancy to avoid further complications and further compromise pregnancy outcomes (NICE 2025). After birth, GDM usually resolves. However, there is a 10‐fold higher risk of developing type 2 diabetes in the mother following GDM (Vounzoulaki et al. 2020). Therefore, healthy eating, regular activity and management of bodyweight are important after birth to reduce the chance of developing type 2 diabetes later in life (Dyson et al. 2018).

### 7.5 Hypertensive Disorders

Hypertension in pregnancy is a common condition, affecting about 10% of pregnant women (Webster et al. 2019). This includes women with chronic hypertension, which may be diagnosed before pregnancy or in the early stages of pregnancy (< 20 weeks' gestation) and women with hypertensive disorders of pregnancy ([HDP], gestational hypertension and pre‐eclampsia).

Gestational hypertension is generally defined as new‐onset hypertension (≥ 140 mmHg systolic or ≥ 90 mmHg diastolic blood pressure) arising after 20 weeks' gestation, whilst preeclampsia is defined as gestational hypertension accompanied by proteinuria (excretion of ≥ 300 mg protein every 24 h) (NICE 2022c). HDP are a major cause of perinatal morbidity and mortality for both mother and offspring.

Factors which have been reported to reduce the risk of HDP include avoiding excessive gestational weight gain, having a high‐fibre diet rich in fruit and vegetables, nuts, whole grain foods, pulses and fish, with adequate vitamin D, calcium and selenium and limiting foods high in fat, salt, sugar and processed red meats (Perry et al. 2022).

A high pre‐pregnancy BMI is positively associated with an increased risk of HDP, including pre‐eclampsia (Perry et al. 2022). Excessive GWG is also associated with a greater risk of developing hypertension (NICE 2025). Therefore, excessive weight gain during pregnancy and between pregnancies should be avoided (Tabet et al. 2021).

### 7.6 Gastroesophageal Reflux Disease (GORD)

Gastroesophageal reflux disease (GORD) is one of the most common problems experienced by pregnant women, reported by 30%–80% of women (NICE 2024). Heartburn, regurgitation and an acidic taste in the mouth are among the most common GORD symptoms, with heartburn and regurgitation causing the most significant negative impact and symptoms tending to increase in severity during the course of pregnancy. It is believed that GORD during pregnancy may be caused by hormonal changes which affect normal gastric motility, increased intra‐abdominal pressure from the growing fetus, slower gastrointestinal transit time or weight gain as pregnancy progresses (Thelin and Richter 2020; Ali et al. 2022).

Lifestyle modifications are initially recommended for those with mild symptoms. These include eating small frequent meals, avoiding foods which trigger symptoms (e.g., spicy, fatty foods and citrus foods), and limiting fluids with meals, instead drinking fluids between meals. Avoiding eating late at night (within 3 h of bedtime), elevating the head of the bed and maintaining an upright posture, especially after eating, and avoiding alcohol and tobacco are also recommended (Ali et al. 2022; Thelin and Richter 2020).

### 7.7 Gingivitis

During pregnancy, many women experience swollen, sore gums that may bleed, a condition known as pregnancy gingivitis. This is caused by a build‐up of plaque on the teeth, which is exacerbated by hormonal changes (Wu et al. 2015). These changes can make gums more vulnerable to plaque, resulting in inflammation, redness and bleeding. Diet plays an important role in supporting good oral health and managing gingivitis during pregnancy. High intakes of free sugars (especially from sugar‐containing drinks) can increase the risk of gum disease and women are advised to limit their intake, especially between meals (NHS 2025).

Some studies have suggested that women with poor gum health are at a higher risk of complications such as preeclampsia, premature birth and low birthweight (Alnasser et al. 2023), possibly because oral infections such as gingivitis have been linked with increased systemic inflammation. However, more research is needed to clarify these associations (Iheozor‐Ejiofor et al. 2017).

A few small studies have indicated that probiotic supplementation might help improve gum health and reduce the severity of gingivitis in pregnant women (Schlagenhauf et al. 2016) and emerging research from Malawi has suggested that chewing sugar‐free gum (gum containing xylitol twice a day throughout the periconception and antenatal period) may have benefits for birth outcomes (Valentine et al. 2024), but research remains limited.

### 7.8 Key Points

- Nausea and vomiting are common, affecting 50%–90% of pregnant women, particularly in the first trimester. Women are advised to consume small, frequent meals and fluids, while avoiding triggers such as specific foods and smells.
- Constipation can occur during pregnancy due to hormonal changes (particularly increased progesterone) that slow intestinal motility. Pregnant women should increase fibre intake (e.g., from wholegrain foods, pulses, fruits and vegetables), stay well hydrated and engage in gentle exercise to prevent occurrence and alleviate symptoms.
- Anaemia can also occur during pregnancy due to expanded plasma volume and insufficient red blood cell production. Iron deficiency is the primary cause, leading to risks such as low birthweight and preterm birth. Iron‐containing foods are recommended (see Tables 3 and 7). Consuming foods containing vitamin C alongside plant sources of iron can enhance absorption. Iron supplements may be prescribed but can cause side effects such as nausea and constipation.
- Gestational diabetes is linked to adverse maternal and fetal outcomes. Management focuses on maintaining blood glucose levels as close as possible to normal through dietary adjustments (e.g., including low glycaemic index carbohydrates and avoiding foods high in sugar) and weight management. Women with GDM have a higher risk of developing type 2 diabetes later in life.
- Hypertension in pregnancy, including gestational hypertension and pre‐eclampsia increases the risk of perinatal morbidity. Diets rich in fibre, fruits, vegetables and certain micronutrients (e.g., vitamin D and calcium) may reduce the risk. Women with a high BMI or excessive weight gain are at increased risk.
- Gastroesophageal reflux disease causes symptoms such as heartburn and regurgitation. Hormonal changes, increased intra‐abdominal pressure and slower gastric transit, contribute to the condition in pregnancy. Lifestyle modifications, such as eating smaller meals, avoiding trigger foods and maintaining an upright posture are recommended for symptom relief.
- Maintaining good oral health is crucial during pregnancy, as hormonal changes can make gums more susceptible to disease. Women should be advised to maintain proper oral hygiene and limit consumption of sugar‐containing foods and beverages, especially between meals.

## 8 Issues for Specific Groups

### 8.1 Vegetarians and Vegans and Other Plant‐Rich Diets

Plant‐based diets are becoming increasingly common but have no formal definition (Kent et al. 2022) and there may be considerable variability in food and nutrient intakes among people who describe their diet as plant‐based, vegetarian or vegan. The term plant‐rich is also used to describe flexitarian diets that emphasise plant‐based foods but still include some animal products. As a result, the nutritional adequacy or inadequacy should not be assumed but assessed based on the specific dietary pattern and supplementation behaviour reported. Although intakes do vary, common forms of plant‐rich diets include:

- **Plant‐rich, flexitarian or semi‐vegetarian diets** primarily emphasise plant‐based foods while still including some animal products, such as dairy and eggs. Individuals following these diets often consume fish and/or poultry more frequently than red meat and typically aim to reduce the overall intake of animal products—especially meat—without eliminating them entirely.
- **Lacto‐ovo‐vegetarians** consume plant and dairy foods and eggs but avoid all meat and fish.
- **Lacto‐vegetarians** consume plant and dairy foods, but avoid all meat, fish and eggs.
- **Vegans** consume only foods of plant origin and exclude all meat, fish, dairy foods, eggs or any foods made from animal products, such as gelatine and suet.

Typically, PBDs can meet the increased demand for energy and protein during pregnancy, although achieving the recommended intake of certain vitamins and minerals may be challenging, especially for strict vegans or those following an organic PBD, due to dietary restrictions and/or avoidance of fortified products (as organic certification does not permit the addition of synthetic vitamins and minerals to food products).

An umbrella review of the health outcomes associated with vegetarian diets identified B12 and zinc deficiencies as the primary concerns among pregnant women consuming PBDs, although few of the included studies specifically assessed this population and those that did are all now at least 10 years old so may not be representative of current PBDs (Niklewicz et al. 2024; Oussalah et al. 2020). Low vitamin B12 status or deficiency was reported in between 17% and 62% of pregnant vegetarians, with higher rates of deficiency associated with longer duration or stricter diet, although functional markers of B12 status were often not assessed. The authors cite a need for further research to understand the implications of low B12 status during pregnancy, proposing a link with detrimental fetal programming and potential future cancer risk and an association with low birth weight and preterm birth (Pawlak et al. 2013, 2014). A meta‐analysis of six observational studies confirmed that pregnant vegetarian women had significantly lower zinc intake than their non‐vegetarian counterparts (Foster et al. 2015).

Maternal and infant cord blood DHA concentrations have been found to be lower in pregnant vegetarians than non‐vegetarians (Lakin et al. 1998). Given the benefits of DHA during pregnancy (see Section 3.3), women following PBDs should be advised to include a reliable source of DHA, whether from fish and fish oil supplements (if including fish in the diet) or vegan microalgae‐based supplements.

Vegan diets have been associated with lower prevalence of excessive GWG in pregnancy (Meulenbroeks et al. 2024), but it has been suggested that vegetarian diets are associated with an increased risk of SGA babies (Kesary et al. 2020; Yisahak et al. 2021; Avnon et al. 2021), mediated by lower pre‐pregnancy BMI, lower pregnancy weight gain or inadequate pregnancy weight gain. However, with adequate food access, infant birthweights and gestational duration are similar in vegetarian and non‐vegetarian pregnancies (Zulyniak et al. 2017; Tan et al. 2019).

The type of dietary advice that is applicable to those following PBDs during pregnancy depends, to a certain extent, on the type of diet followed. As well as considering the types and quantities of animal products included in the diet, it is essential to assess whether fortified plant‐based alternatives are intentionally avoided, as in the case of an organic diet, and whether any supplements are taken. Fortified foods can be an important source of vitamin B12 for individuals who do not consume animal products and may also contribute to iodine in the UK diet. Supplements are often a key dietary source of vitamin D and omega‐3 fats for both vegetarians and non‐vegetarian consumers.

Table 7 lists suitable, alternative food sources for nutrients that may be inadequate in some vegetarian, vegan and plant‐based diets and that are important during pregnancy.

**TABLE 7:** Foods suitable for vegan and plant‐based diets providing important nutrients during pregnancy.

At risk nutrient	Vegan sources	Lacto‐ovo vegetarian or pescatarian diets	Comments
Iron	Pulses, dark green leafy vegetables, wholemeal bread and fortified foods including white bread and iron fortified breakfast cereals, dried fruit, nuts and seeds	Eggs Some types of fish (e.g., sardines, mackerel, tuna, salmon)	Bioavailability of non‐haem iron, as found in plant foods, is lower than haem iron (only found in animal sources). Absorption inhibited by tannins (e.g., tea), phytates (e.g., cereals and pulses), oxalates and fibre. Absorption of non‐haem iron promoted by vitamin C containing foods (see Section 5.5.7)
Calcium	Green leafy vegetables (e.g., kale, watercress, bok choy, calcium‐set tofu) and calcium fortified foods (e.g., bread and soya drinks), nuts and dried fruit	Milk and dairy products. Fish containing soft bones	Most plant‐based dairy alternatives are fortified. Bioavailability of plant sources, can be lower than dairy foods
Vitamin B12	B12 fortified foods (e.g., yeast extract, soya protein, plant‐based dairy alternatives and breakfast cereals)	Dairy products, eggs	If consuming organic/unfortified products, a supplement is advised
Zinc	Whole grain foods (e.g., wholegrain and seeded breads and breakfast cereals), nuts and seeds	Cheese	Phytates (from cereals and pulses) inhibit zinc absorption, although innovative biofortification and agronomic fortification are seeking to address
Iodine	Plant‐based dairy alternatives (if fortified with iodine)	Cows' milk and other dairy foods (e.g., cheese and yogurt), fish, especially white (e.g., cod, haddock and pollock) and some shellfish (e.g., prawns, crab and mussels), eggs	Ensure plant‐based dairy alternatives are fortified. Caution with seaweed consumption (see Section 4.2.3)
Omega‐3 fats	ALA in linseed (or flaxseed), rapeseed oil, walnut oil, soyabean oil and blended vegetable oils, tofu and walnuts Some seaweeds. Algal DHA supplements.	Omega‐3 fortified foods (e,g., eggs)	Levels added to foods may be low. ALA to DHA/EPA conversion is limited. Caution with seaweed consumption (see Section 3.3)
Vitamin D	Vitamin D fortified foods including fat spreads, breakfast cereals, soya milk and other soya products. D2 (plant source derived) supplements or vegan vitamin D3 from lichen	Oily fish, dairy products, eggs, D3 supplements (typically derived from lanolin found in sheep's wool)	Few natural sources of vitamin D. Levels added to foods may be low. Skin production via safe sun exposure. Consideration of supplementation (10μg/day) during the Autumn/ Winter months in the United Kingdom (see Sections 4.2.1 and 5.5.5)

Abbreviations: ALA, alpha linolenic acid; D2, ergocalciferol; D3, cholecalciferol; DHA, docosahexaenoic acid; EPA, eicosapentaenoic acid.

### 8.2 Teenage Pregnancy

The number of teenage pregnancies in the United Kingdom has fallen from ~100 000 per year in the 1990s to just over 40 000 in 2020 (ONS 2023). Approximately 8% of UK births were to mothers under the age of 20 years in 1990, which fell to less than 3% by 2020 (WHO 2024). Teenage pregnancy is typically associated with lower GWG, increased risk of low birth weight, pregnancy‐induced hypertension, iron‐deficiency anaemia, preterm labour and maternal mortality (Felice et al. 1999). However, it is possible that this could represent confounding factors such as pre‐pregnancy BMI, length of gestation, ethnicity, smoking and alcohol intake (Goldberg 2002).

The NDNS data suggest that girls aged 11–18 years in the United Kingdom have a particularly high prevalence of nutrient intakes below the LRNI (Table 8); (OHID 2025). Specifically, the proportion of girls aged 11–18 years with dietary intakes below the LRNI for vitamin A, riboflavin, calcium, iron, iodine, magnesium, potassium and zinc tends to be greater than for women aged 19–64 years. Furthermore, there is evidence that for some of these nutrients, the proportion of girls aged 11–18 years with dietary intakes below the LRNI has been increasing. This is particularly evidenced for iodine where time trend analysis indicates a decrease in urinary iodine concentrations in this age group of 29% between 2013 and 2023 (OHID 2025). These findings are supported by blood biomarker data (Table 9), which show that girls aged 11–18 years are more likely to have levels below thresholds, particularly for red cell folate and ferritin, compared to women aged 19–64 years and girls of the same age in 2008‐10.

**TABLE 8:** Proportion of girls 11–18 years and women 19–64 years with intakes below the LRNI, NDNS 2019 to 2023.

Nutrient	Girls aged 11–18 years (%)	Women aged 19–64 years (%)
Vitamin A	18	8
Riboflavin	32	19
Folate	9	9
Calcium	18	8
Iron	49	34
Iodine		
Magnesium	48	15
Zinc	23	7

**TABLE 9:** Proportion of girls aged 11–18 years and women aged 18–65 years with low blood status of markers of selected nutrients.

Parameter	Girls aged 11–18 years (%), 2008–2010	Girls aged 11–18 years (%), 2019–2023	Women aged 19–64 years (%), 2019–2023
Haemoglobin			
< 120 g/L	7	11	4
Ferritin			
< 15 mg/L	20	28	9
Haemoglobin <120 g/L and ferritin < 15 mg/L	3	9	1
Red cell folate			
< 350 nmol/L	1647	33	8
< 450 nmol/L		60	25
Serum folate			
< 10 nmol/L	2445	52	3862
< 15 nmol/L		80	
25‐OHD*			
< 25 nmol/L	18	26	17

*Source*: OHID 2025; PHE, 2020a. *25‐OHD: 25‐hydroxy vitamin D.

The prevalence of low micronutrient status could be a combination of both lower micronutrient intakes and increased micronutrient requirements, with potential additional competition for nutrients for growth and development of both the mother and the fetus (Black et al. 2013). It is possible that maternal growth and development is compromised to preserve the growth and development of the fetus. A higher GWG is therefore recommended for adolescent pregnancies in the United States (IOM 1990). It has also been suggested that there may be reduced fetal nutrient availability during teenage pregnancy due to immature placental development (Goldberg 2002), thus further emphasising the importance of maternal nutrient status.

Calcium is a nutrient of particular importance during teenage pregnancy due to the rapid increase in bone mass during adolescent years as the maternal skeleton is developing (see Section 5.5.6). Whilst some physiological adaptations can assist in meeting the additional calcium requirement during pregnancy, teenage girls require more calcium than adults for skeletal development. The UK RNI for calcium for girls aged 11–18 years is 800 mg/day, compared with 700 mg/day for adult women (COMA 1991). Therefore, the relatively high prevalence of girls aged 11–18 years with calcium intakes below the LRNI is due to both a lower absolute intake than adult women (median intake 650 vs. 695 mg/day) (OHID 2025) and a higher requirement.

Almost half of teenage girls display iron intakes below the LRNI in the United Kingdom, with a high proportion also displaying low iron stores as indicated by low serum ferritin. This has been attributed to low iron intakes from diet composition, low energy intake and/or vegetarian and vegan diets, combined with growth and onset of menstruation. Maternal iron deficiency anaemia is associated with preterm delivery and subsequent low birth weight (see Section 7.3). Therefore, adequate iron intake throughout pregnancy, especially in teenagers, is of importance. Iron supplements may be prescribed if necessary.

Folate intake is of particular concern during pregnancy since inadequate folate intake is associated with an increased risk of neural tube defects in the fetus (see Section 4.2.2). Accordingly, folic acid supplements are recommended before conception and until the 12th week of pregnancy. This becomes a particular problem in teenage pregnancy since it is often unplanned; prior estimates are that 75% of teenage pregnancies are unplanned (Goldberg 2002). Therefore, supplementation prior to conception and in early pregnancy is often compromised either because the pregnancy is unplanned or the importance of supplementing with folic acid is unknown. Moreover, irregular menstruation is common in adolescence and can limit pregnancy detection during the early stages where folate is important. Some teenage girls may also be particularly preoccupied with body weight and/or may restrict weight gain in an attempt to conceal pregnancy. This may further compound nutrient inadequacies. Indeed, energy intake restriction during pregnancy has been associated with an increased risk of neural tube defects (Carmichael et al. 2003).

Maternal iodine status is associated with cognitive development of offspring (see Section 4.2.3) and it is notable that not only do teenage girls display a greater prevalence of low iodine intake than adult women (Table 8), but the prevalence of low iodine intake has increased from 2008/2010 to 2019/2023 (OHID 2025), possibly due to a shift in diets away from the primary sources of iodine such as cow's milk and changes in farming practices.

Teenage pregnancy therefore presents additional nutritional challenges to health professionals, with increased nutrient requirements and sometimes lower nutrient intakes in relation to key micronutrients required for fetal development. Therefore, good dietary advice and support for teenagers who become pregnant is important to support the mother through adolescent development in addition to optimising the growth and development of the fetus.

### 8.3 Multiple Pregnancies

Multiple pregnancies (the presence of more than one fetus in the womb) increase metabolic demands, leading to faster depletion of maternal nutrient reserves compared to a singleton pregnancy (Zgliczynska and Kosinska‐Kaczynska 2021; Shinagawa et al. 2005). Women pregnant with twins typically gain more weight than those with a singleton pregnancy and are at higher risk of adverse pregnancy outcomes (Schubert et al. 2023). Infants from multiple pregnancies have also been shown to have lower DHA status compared to those from singleton pregnancies (see Section 3.3).

NICE recommends that women carrying twins or triplets receive the same advice on diet, lifestyle and nutritional supplements as women with singleton pregnancies (NICE 2024). However, the incidence of anaemia is higher in women with a twin or triplet pregnancy and so iron or folic acid supplementation may be required (NICE 2024). Some studies have also found lower vitamin D status in women with multiple gestations compared to those with singletons (Zgliczynska and Kosinska‐Kaczynska 2021). Further research is needed to better understand the nutritional adjustments required for women with multiple pregnancies.

### 8.4 Smokers

Women are advised not to smoke before conception and while pregnant as smoking during pregnancy is known to have adverse effects on birth outcomes, including increased risks of low birthweight, stillbirth, miscarriage and preterm delivery (Avşar et al. 2021). Women who smoke during pregnancy may be at increased risk of poor nutritional status, increasing the risk of inadequacies of important nutrients that may impact both mother and the developing fetus. Studies have shown links between smoking and reduced levels/status of micronutrients in mothers (O'Malley et al. 2018) and their offspring, for example decreased iron stores in babies (Pateva et al. 2015). The diets of smokers are generally poorer than non‐smokers (Alruwaili et al. 2024; Corrales‐Gutierrez et al. 2022). Some studies have also reported adherence to folic acid supplementation to be lower (Corrales‐Gutierrez et al. 2022). In England, 9.5% of women are recorded as smoking at the time of delivery (as of 2020/21) (NHS England 2021). Rates of smoking in pregnancy have a strong social and age gradient with poorer and younger women much more likely to smoke in pregnancy (PHE 2019b).

### 8.5 Ethnic Minority Groups

Ethnic minority groups (those with different national or cultural traditions from the main population) can be more vulnerable in terms of nutrition during pregnancy, due to a variety of factors. These include socio‐economic inequalities, cultural differences, more limited access to healthcare and higher rates of certain health conditions. For example, in the United Kingdom, South Asian and Black African‐Caribbean populations are at higher risk of gestational diabetes (Schoenaker et al. 2023; Garcia et al. 2021), high blood pressure (preeclampsia) (Raphael et al. 2024), obesity and excessive GWG (Raju et al. 2024), all of which can impact on birth outcomes and longer‐term health outcomes for the child. Rates of preterm birth in the United Kingdom are also higher in women from Asian or black ethnic groups than in women from white ethnic groups (Kayode et al. 2024).

Additionally, some ethnic minority groups are more prone to micronutrient inadequacies. For example, those with darker skin (such as South Asian, African and Caribbean communities) living in the United Kingdom are more likely to have lower levels of vitamin D during pregnancy (Curwain et al. 2021). This vitamin is crucial for maternal bone health and immune function (see Section 5.5.5). The higher risk of vitamin D deficiency in these populations is often exacerbated by cultural factors such as wearing clothing that covers the skin and spending less time outdoors, which can limit the body's ability to produce vitamin D naturally. South Asian women, particularly those from Pakistani or Bangladeshi backgrounds, have been shown to be less likely to take folic acid supplements (see Section 4.2.2) (Schoenaker et al. 2023; Brough et al. 2009). These issues can be exacerbated by more limited access to healthcare in relation with a lack of culturally sensitive dietary information. This highlights the need for targeted, co‐created resources and public health interventions that provide culturally appropriate nutritional support, promote prenatal supplementation and address the particular challenges faced by these communities (Raju et al. 2024).

### 8.6 Key Points

- Plant‐rich diets, including flexitarian, vegetarian and vegan diets, vary widely in terms of food and nutrient intake and their adequacy during pregnancy should be evaluated based on individual dietary patterns, supplementation and food choices. However pregnant women following some of these diets may struggle to meet recommended levels of certain nutrients. For example, strict vegans may have lower intakes of important nutrients such as vitamin B12, DHA and iodine and supplementation or fortified sources may be required.
- Teenage pregnancies are associated with higher risks of low birth weight, preterm labour and complications such as hypertension and anaemia. These risks may be influenced by factors such as pre‐pregnancy BMI and lifestyle choices.
- Adolescent girls (aged 11–18 years) in the United Kingdom have higher rates of nutrient inadequacies, particularly in calcium, iron, iodine and folate. Increased requirements of these nutrients during pregnancy can therefore pose a significant challenge and uptake of supplements, for example folic acid supplements before conception, is relatively low in this age group. Targeted nutritional support and supplementation for pregnant teenagers are essential to ensure optimal growth and development for both mother and fetus.
- Multiple pregnancies increase metabolic demands, leading to faster depletion of maternal nutrient reserves, higher weight gain and increased risks of maternal anaemia and lower vitamin D. In the United Kingdom, NICE recommends the same dietary and supplementation advice for women with twins or triplets, though additional supplements may be required on a case‐by‐case basis, depending on individual nutritional needs.
- Smoking during pregnancy is linked to a range of adverse birth outcomes and women who smoke are at an increased risk of poor nutritional status. Smokers often have poorer quality diets and lower adherence to folic acid supplementation. Higher smoking rates are observed among younger and lower socio‐economic groups.
- Ethnic minority groups in the United Kingdom, particularly South Asian and Black African‐Caribbean populations, have higher rates of preterm birth and are at higher risk for pregnancy‐related health conditions such as gestational diabetes, high blood pressure (preeclampsia), obesity and excessive weight gain, which can impact birth outcomes and long‐term child health. Women from these groups are more prone to vitamin D deficiency due to cultural factors and may be less likely to take prenatal and antenatal supplements (e.g., prenatal folic acid). Barriers including limited healthcare access and a lack of culturally sensitive information exacerbate such nutritional vulnerabilities during pregnancy.

## 9 Physical Activity

Regular physical activity confers a net health benefit for almost all populations including pregnant women and is therefore a regular component of health guidelines. Physical activity can affect almost every physiological system and therefore has clear potential to influence nutritional requirements during pregnancy (Ashcroft et al. 2024). The effects of physical activity on nutritional requirements can be direct or indirect. During physical activity, there is an increase in energy expenditure, which is largely met by carbohydrate and fat oxidation. The increase in energy expenditure is also accompanied by increases in heart rate, blood pressure and substantial changes in blood flow. Therefore, there is a direct consequence of increasing physical activity on energy requirements (Ainsworth et al. 2011). An indirect (theoretical) benefit of energy balance is maintained, is that a varied diet consumed at a higher energy expenditure will result in a higher intake of micronutrients, potentially lowering the chances of nutrient deficiency if the metabolism and utilisation of those nutrients remains relatively stable.

When performed over a period of weeks, physical activity results in a variety of physiological adaptations including increased plasma volume, haemoglobin mass and skeletal muscle mitochondrial content (Ainsworth et al. 2011). These adaptations seem to persist during pregnancy, since physically active pregnant women display higher estimated plasma and red cell volumes compared with women who are not as physically active (Pivarnik et al. 1994). Furthermore, maintaining high levels of physical activity throughout pregnancy has been shown to increase villous volume of placental tissue, potentially enhancing placental efficiency for gas and nutrient exchange (Jackson et al. 1995). The impact of these adaptations on nutritional requirements is not fully known. Theoretically, further increases in red cell mass from physical activity during pregnancy may increase iron requirements, especially during the dynamic phase of red cell synthesis and could be exacerbated by weight‐bearing exercise due to intravascular haemolysis. Increases in carbohydrate and fat oxidation rates and skeletal muscle protein turnover from regular exercise may also increase dietary carbohydrate, fat and protein requirements. Objective evidence to adequately inform guidelines for the physically active pregnant woman, however, is currently lacking.

At present, the UK Chief Medical Officer's guidelines recommend that pregnant women aim to accumulate at least 150 min of moderate‐intensity aerobic activity spread throughout the week (DHSC 2019). This could include activities such as brisk walking, swimming or stationary cycling. In addition to aerobic exercise, NICE suggests that pregnant women should engage in muscle‐strengthening activities on two or more days per week (e.g., exercises using bodyweight, resistance bands or light weights) as well as daily pelvic floor exercises to help prevent urinary incontinence. Activities with a high risk of falling or abdominal injury, such as contact sports, high‐impact sports, skiing, horseback riding and scuba diving, should be avoided and pregnant women should consult a healthcare provider before starting an exercise regime, especially if they have any medical conditions or pregnancy complications. Staying well‐hydrated during exercise is also important.

### 9.1 Key Points

- Regular physical activity during pregnancy increases energy expenditure, which directly impacts energy needs. It also leads to physiological adaptations such as increased plasma volume and red cell mass, which may affect nutritional needs, particularly for iron, carbohydrates, fats and proteins.
- Physical activity improves overall health and has clear benefits for pregnant women, including better placental function, higher blood volume and increased exercise tolerance. The UK guidelines recommend at least 150 min of moderate‐intensity aerobic activity per week, along with muscle‐strengthening exercises and pelvic floor workouts.

## 10 Capitalising on Opportunities for Targeted Dietary Support and Guidance During Pregnancy

This review underscores the essential role of diet in supporting maternal and fetal health during pregnancy. Women require clear guidance on a range of dietary and lifestyle factors, including nutritional needs, supplementation, food safety, physical activity and weight management (Table 10). Pregnancy is a crucial time for the development of obesity in later life, due to factors such as excessive GWG and failure to return to pre‐pregnancy weight (Berezowsky and Berger 2021), as well as its role in intergenerational obesity development (Heselhurst et al. 2019). A healthy diet, typically characterised by high intakes of vegetables, fruits, whole grains, pulses, lower‐fat dairy foods and lean proteins, alongside limiting processed meats and foods high in saturated fats, salt and sugar is crucial to ensuring that both mother and baby thrive throughout the pregnancy and beyond. Adherence to healthier dietary patterns, both before and during pregnancy, has been linked to improved micronutrient intake (Tanha et al. 2013), prevention of excessive GWG (Muktabhant et al. 2015) and a number of better maternal and offspring outcomes (Chia et al. 2019; Raghavan et al. 2019; Lecorguillé et al. 2023; Mou et al. 2024).

**TABLE 10:** Summary of key diet‐related recommendations for women in the United Kingdom to support a healthy pregnancy.

Category	Pre‐pregnancy	During pregnancy
General advice	A balanced and varied diet can provide the necessary vitamins and minerals to support fertility and a healthy pregnancy (along with supplements where needed, see Table 11). In particular, foods containing **vitamin D** (e.g., oily fish, eggs and vitamin D fortified breakfast cereals); **folate** (e.g., green leafy vegetables, wholegrain bread, fruits like oranges and berries, fortified breakfast cereals); **iodine** (e.g., dairy products and iodine‐ fortified alternatives, fish, shellfish and eggs); **calcium** (e.g., dairy foods and fortified alternatives, green leafy vegetables, canned fish and breads) and **iron** (e.g., red meat, fish, eggs, pulses, nuts, seeds, dried fruit, fortified breakfast cereals) should be included in the diet (see Sections 4 and 5)	Pregnancy provides a great opportunity for women to modify their diet and make changes to benefit the health of themselves and their child. Contact with health professionals is an opportunity to discuss diet and lifestyle issues. Women should be advised to continue to consume a nutrient‐dense diet (as for pre‐pregnancy) as requirements rise for many micronutrients including thiamine, riboflavin, folate, vitamin C, vitamin A and iodine (see Section 5). Requirements for the long chain omega‐3 fatty acid DHA also rise during pregnancy, which is found predominantly in oily fish (see Section 3.3 for other sources and 6.5 for safety advice)
Weight	Extremes of body fat and weight can impair fertility in both men and women. Achieving and maintaining a healthy weight (BMI 18.5–24.9 kg/m^2^) prior to conception is recommended. For women living with obesity (BMI ≥ 30 kg/m^2^), weight reduction is advised (see Section 4.1)	Maternal obesity is associated with an increased risk of adverse pregnancy outcomes, including gestational diabetes, type 2 diabetes and cardiovascular disease. Women may seek guidance on weight management during pregnancy, particularly if they gained excessive weight in previous pregnancies. While restrictive dieting is not recommended, avoiding excessive gestational weight gain is important for maternal and fetal health (see Sections 2.1 and 5.1)
Diet quality	Pre‐pregnancy nutritional status is key. Women should be advised to consume a healthy and varied diet (as depicted by the Eatwell Guide): including fruit and vegetables, wholegrain and other fibre rich‐foods, alongside iron and vitamin B12‐rich foods, fish, some dairy products (or calcium and iodine fortified alternatives) and sources of healthy fats (e.g., from nuts, seeds, avocados, oily fish) (see Section 4.2)	A healthy, balanced diet continues to be key to provide the nutrients required during pregnancy. Pregnant women are encouraged to follow the Eatwell Guide recommendations (see Section 5)
Specific nutrients	Folic acid (400 μg/day) is recommended for those trying to conceive to reduce risk of NTDs. Adequate iodine and vitamin D intake are important and supplements should be considered from October to March for all and throughout the year for those at ‘high risk’ of deficiency (see Table 11 and Section 4.2.2)	During pregnancy, folic acid (400 μg/day) is recommended for the first 12 weeks and women are advised to consider supplementation with vitamin D from October to March and throughout pregnancy for those at high risk (see Table 11 and Section 5.5.5). Additional DHA is needed from the second trimester through dietary sources (e.g., oily fish) or supplements but supplements containing retinol should be avoided (e.g., cod liver oil) (see Section 5.3)
Plant‐based diets	Those following a plant‐based diet (especially a vegetarian or vegan diet), should be advised to consume adequate intakes of vitamin B12, zinc, iodine, omega‐3 fats and iron through nutrient‐rich plant‐based foods, fortified foods and supplements where needed (see Section 8.1)	Advice as for pre‐pregnancy (see Section 8.1)
Fluids	Women should be encouraged to drink at least six to eight glasses of fluid per day. Water is the best choice and sugar‐sweetened options should be limited	Regular fluid intake (at least eight 200 mL glasses/day) will support adequate hydration (see Section 5.6). It is recommended to limit intakes of fizzy drinks or other drinks that are high in sugar. Water, milk and no‐added‐sugar fruit juices (limited to 150 mL per day) are good choices to support both hydration and nutritional needs. Caffeine intake should not exceed 200 mg/day (see Table 5) and alcohol is best avoided entirely. Energy drinks can contain high levels of caffeine and other ingredients that may be harmful during pregnancy and should be avoided. Up to 1–2 cups of herbal and green tea is safe but high intakes of some herbs are not advised (some herbal teas may also contain caffeine)
Food safety	Including fish in the diet is a healthy choice. However, for women planning a pregnancy there are recommendations about avoidance/maximum intakes for certain types of fish, because of the levels of mercury and pollutants that some fish can contain. Current advice is to not to eat shark, swordfish and marlin and to limit oily fish to no more than two portions (each 140 g cooked weight), no more than four cans of tuna a week or no more than two tuna steaks a week (see Section 6.5)	To avoid foodborne illness, women should choose foods with low risk of food poisoning and practice good food hygiene (see Section 6). High‐risk foods include unpasteurised milks and foods made from them (e.g., soft ripened goats' cheese), mould‐ripened cheeses (unless steaming hot), raw/undercooked meats, liver and liver products, pâtés, game meats (especially if shot with lead), raw or partially cooked eggs (unless British Lion/Laid in Britain), raw shellfish, cold‐smoked or cured fish (unless steaming hot). Eating fish is recommended during pregnancy but oily fish should be limited to 2 portions per week (each 140 g cooked weight) and tuna to no more than two steaks or four medium‐sized cans per week, with shark, swordfish and marlin avoided as these contain high levels of mercury. Liquorice root should be avoided. Seaweed is not recommended to be eaten more than once a week during pregnancy.
Other lifestyle factors	General lifestyle advice includes not smoking, being active on most days (at least 150 min of moderate intensity aerobic activity per week (e.g., brisk walk) or 75 min of vigorous exercise per week (e.g., running) along with muscle strengthening exercises at least twice a week), stress management and adequate sleep	General lifestyle recommendations remain: no smoking, regular physical activity (at least 150 min of moderate‐intensity aerobic activity per week, along with muscle‐strengthening exercises and pelvic floor workouts, avoiding any strenuous exercise in hot weather, being cautious with activities with a high risk of falls or abdominal injury and avoiding contact sports and scuba diving), stress management and adequate sleep

*Note*: Based on advice from NHS Choices (NHS 2023a, 2023b).

Emerging evidence also suggests that maternal diet during pregnancy and lactation may influence early food acceptance in infants. Limited but consistent evidence indicates that certain flavours (e.g., garlic, carrot and kale) can pass from the maternal diet during pregnancy into the amniotic fluid, with some studies showing that fetal flavour exposure may increase acceptance of similarly flavoured foods during infancy, although any impact on childhood dietary intake remains to be established (Spill et al. 2019; Ustun‐Elayan et al. 2025).

Ensuring good nutritional status prior to conception is essential to build the necessary nutrient reserves and avoid the need to make significant dietary changes during pregnancy. However, motivation to improve diet may be low before pregnancy for some women, particularly those in hard‐to‐reach groups (e.g., low socio‐economic groups) who are at highest risk for poor pregnancy outcomes (Langley‐Evans et al. 2022). In contrast, the antenatal period provides a unique opportunity for intervention, as women are often highly receptive to health messages and in frequent contact with healthcare professionals creating a critical window for dietary guidance (Thangaratinam et al. 2012; Phelan 2010). Structured lifestyle support has been shown to improve maternal diet, increase physical activity and reduce excessive weight gain (Barker et al. 2016).

Despite this potential, many women, before and during pregnancy, fall short of the dietary targets outlined in official guidelines (see Section 5), highlighting the need for targeted and effective dietary support, particularly for the most vulnerable groups. For example, women in food‐insecure or low‐income households are at higher risk of inadequacies in key nutrients such as iron, vitamin D, calcium and folate (Murphy et al. 2022; PHE 2019a), whilst adolescents or those with multiple pregnancies have higher nutritional needs, further complicating the challenge of meeting dietary guidelines (see Sections 8.2 and 8.3). Studies indicate that many pregnant women have limited awareness of dietary guidance and inadequate access to nutritional advice during pregnancy (Swift et al. 2017; McCarthy et al. 2024).

**FIGURE 3:**
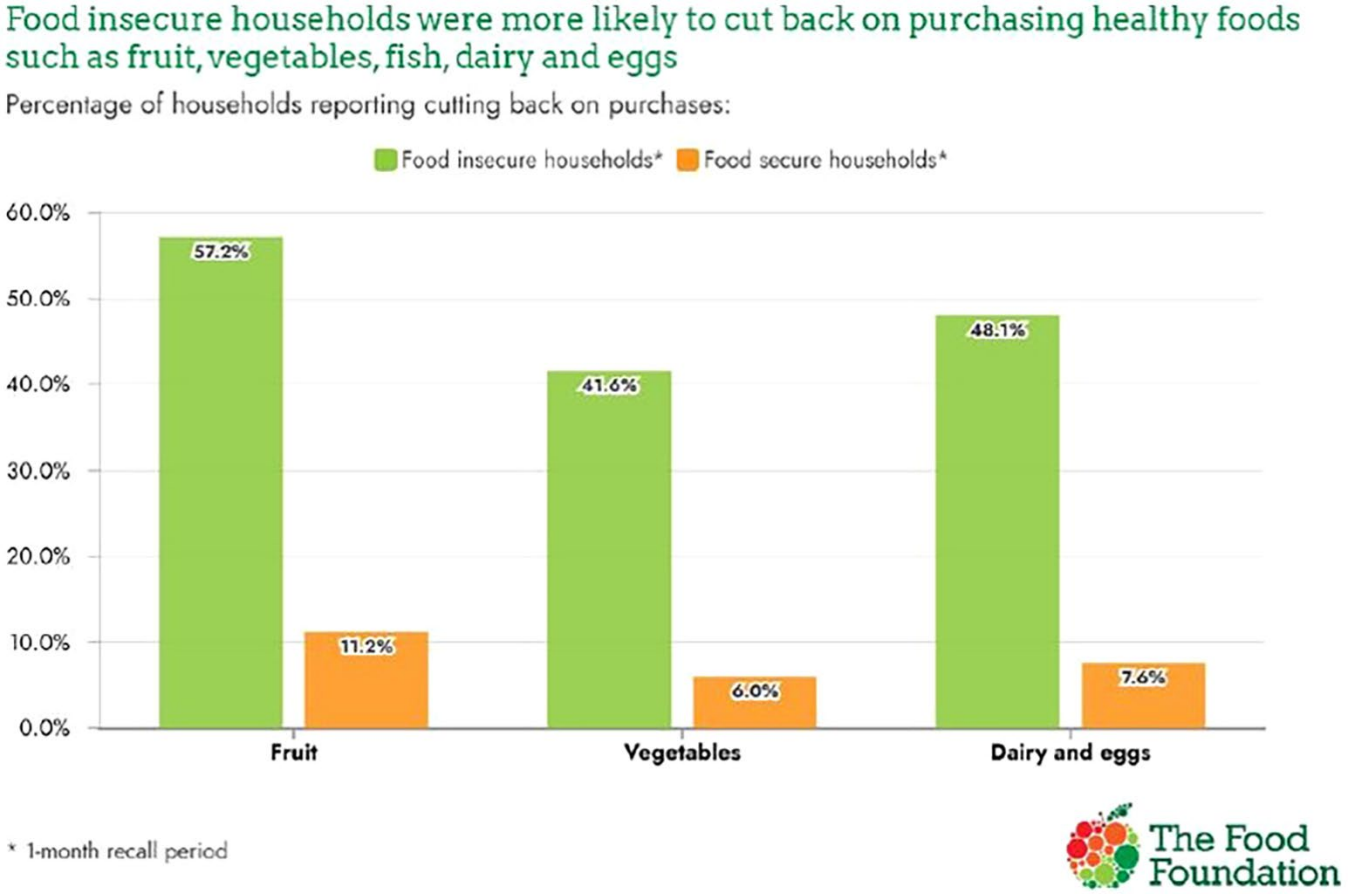
Percentage of households reporting cutting back on purchases of specific foods (January–February 2025). Based on 1‐month recall period. *Source*: Food Foundation (2025).

The ongoing cost‐of‐living crisis has exacerbated food insecurity, forcing many women to rely on cheaper, energy‐dense foods that are often low in micronutrients, thereby increasing nutrient gaps. In a survey by the Food Foundation in 2025, 57% of food insecure households reported eating less fruit and 42% buying fewer vegetables, as well as less fish, dairy and eggs (Figure 3; Food Foundation 2025). Systematic reviews have reported associations between food insecurity among women and reduced intakes of energy and nutrients critical for preconception and pregnancy including vitamin A, vitamin B6, calcium, magnesium, zinc and folate (Nguyen et al. 2024). Food insecurity also impacts maternal weight (Nguyen et al. 2024) and contributes to higher levels of stress and anxiety, which can negatively affect eating behaviours and appetite, further compounding the issue of inadequate nutrition (Augusto et al. 2020).

Nutrient supplementation also plays an important role, especially for women who struggle to meet all their nutritional needs through diet alone (Table 8). For instance, folic acid is vital for reducing the risk of neural tube defects. However, studies suggest compliance with folic acid supplementation or dosage to be inadequate among many women, particularly those in lower‐income households where cost can be a significant barrier (Stockley and Lund 2008), women living with obesity (Linnell et al. 2022), those with higher parity (Moser et al. 2019) and women in some ethnic minority groups (Brough et al. 2009; Schoenaker et al. 2023).

These challenges highlight the importance of improving access to both nutritious food and supplementation, particularly for women facing food insecurity. Without proper support, women in these circumstances are at greater risk of adverse pregnancy outcomes, such as preterm birth, low birthweight and developmental delays. In England, Wales and Northern Ireland, free prenatal vitamins alongside monetary support to purchase nutrient‐dense foods such as milk, fruit and vegetables and pulses are available to women under 18 or those receiving means‐tested benefits such as Universal Credit or Income Support, through the Healthy Start Scheme ( gov.uk 2025). In Scotland, Best Start Foods also offers a grant towards healthier foods for pregnant women on certain benefits or under the age of 18 (mygov.scot 2024) and universal access is provided to Healthy Start supplements (Scottish government 2024). However, take‐up of these schemes is lower than expected and many barriers to uptake, particularly of the prenatal supplements, have been identified such as low maternal awareness, limited access in geographically isolated (particularly rural) regions (Barrett et al. 2024; Which? 2025) and health professionals' mixed messaging or disengagement with the schemes (Moonan et al. 2022). Additionally, for ethnic minority groups, there may be further challenges related to culturally sensitive information. For example, cultural differences, language barriers and limited trust in healthcare systems may result in lower engagement with prenatal care, including the use of supplements such as folic acid and iron. Streamlining access to prenatal supplementation and improving awareness of the schemes and other available resources, as well as ensuring health information is culturally appropriate, accessible and delivered in a way that resonates with the diverse communities being served, is essential to ensure that no woman is left behind in receiving vital support during pregnancy.

Women's ability to adopt healthier behaviours during pregnancy is often constrained by time pressures, financial limitations, work and caregiving responsibilities and lack of flexible healthcare services. These structural barriers, particularly in underserved or marginalised groups, can limit both engagement with dietary advice and sustained behaviour change. Generic dietary advice is less effective than dietary support that is personalised, practical and culturally sensitive, considering nutritional needs, food preferences, cultural background, health literacy, access to food and personal beliefs (Chen and Antonelli 2020; Jinnette et al. 2020). Interventions incorporating behaviour change techniques—such as goal setting, action planning and self‐monitoring—and delivered through trusted professionals or peer networks in flexible formats (e.g., digital, group‐based or community‐led approaches) are more likely to improve dietary quality and adherence (O'Connor et al. 2025).

Pregnancy and the broader periconceptional window, offers a ‘teachable moment’ when many women are especially open to adopting health‐promoting behaviours (Barker et al. 2016; Langley‐Evans et al. 2022). However, this opportunity is easily missed without effective communication strategies. At the same time, willingness to seek advice and information can result in women accessing other sources which are not reliable (Langley‐Evans et al. 2022). There is also a growing opportunity to integrate sustainable eating practices into prenatal care. Many women are increasingly aware of the environmental impact of their food choices (FSA 2021), providing a timely opportunity to encourage sustainable practices that benefit both maternal health and the environment. This shift offers a chance to promote sustainable dietary habits, such as incorporating more plant‐based foods and adopting a dietary pattern that more aligns with the Eatwell Guide, which could benefit both the mother's health and the environment (Scheelbeek et al. 2020). However, as dietary patterns shift towards more plant‐rich diets, they can present further challenges in ensuring adequate intake of key nutrients typically found in animal products, such as vitamin D, iodine, iron, calcium and vitamin B12 (Neufingerl and Eilander 2021). Pregnant women should be encouraged to diversify their plant‐based food choices and incorporate fortified foods, such as plant‐based alternatives enriched with calcium, vitamin D, iodine and B12 and advised on appropriate use of supplements where appropriate (see Table 11) to help fill potential nutrient gaps.

**TABLE 11:** Current UK advice for supplementation during pregnancy, including dosage and timing.

Nutrient	Population sub‐groups (if applicable)	Required intake (daily)	Timing	Comments
Folic acid		400 μg	At least 3 months pre‐conception and for first 12th weeks of pregnancy	Included in Healthy Start (Sections 4.2.2 and 5.5.2)
	Increased risk of NTD (those with previous NTD affected pregnancy, couples where either has a family history of congenital malformations, women with diabetes or a haematological condition requiring folic acid supplementation or taking medication affecting folic acid absorption)	5 mg^a^	At least 3 months pre‐conception to 12th week of pregnancy	Requires a prescription (Sections 4.2.2 and 5.5.2)
Vitamin D		10 μg	From October to March	Included in Healthy Start vitamins (Section 5.5.5)
	Women at high risk, that is, those with dark skin, those who cover their skin or spend lots of time indoors	10 μg	Throughout pregnancy	Included in Healthy Start vitamins (Section 5.5.5)
Vitamin A	Women taking supplements containing retinol or eating foods rich in vitamin A (e.g., liver)	Avoid high intakes (daily requirement 700 μg)	Throughout pregnancy	Daily requirement achievable through diet. Avoid supplements containing retinol (e.g., cod liver oil) (Sections 5.5.1 and 6.1)
DHA/EPA		450 mg	From 20 weeks onward	Supplements are available but can be expensive. Recommended intake is achievable by regularly eating oily fish (up to twice a week) (Sections 3.3 and 5.3)
	Low DHA intakes or status^a^	600–1000 mg	From 20 weeks onward	Dose only achievable from supplements (Section 3.3)
Iron	Low iron stores or iron deficiency anaemia^a^	14.8 mg	Throughout pregnancy	As required—prescribed by GP (Section 5.5.7)
Iodine	Those not consuming fish or dairy foods	150 μg	Preconception	Supplemental iodine should be in the form of potassium iodide or potassium iodate. Kelp or other seaweed‐based supplements are not recommended (Sections 4.2.3 and 5.5.8)

^a^ Should be provided with advice from health professional.

Despite the clear need for comprehensive nutrition‐related support during pregnancy, healthcare professionals often face challenges in addressing dietary, food safety and weight‐related concerns (Lee et al. 2012; Beasant et al. 2023; Wennberg et al. 2014; Arrish et al. 2017). In particular, some report feeling uncertain or uncomfortable discussing weight management, often due to a lack of training or fear of causing distress (Heslehurst et al. 2014). Yet, women themselves express a desire for more support in these areas, including weight management (Nolan et al. 2024). It is crucial to empower healthcare professionals with the training, tools and support they need to engage in these conversations effectively and sensitively, especially if partners are also present (Walker et al. 2019; NICE 2025).

Providing healthcare professionals with the right resources will ensure they are well‐equipped to help women navigate their nutrition, supplementation, lifestyle and weight management needs, ultimately optimising maternal and fetal health and reducing the risks of adverse outcomes. Such timely and effective dietary and lifestyle counselling could help women adopt healthier habits that benefit both the pregnancy and the long‐term health of their children, potentially breaking the cycle of poor health and improving public health outcomes across generations.

### 10.1 Key Points

- A healthy diet before and after conception is essential for both maternal and fetal health and should include high intakes of vegetables, fruits, pulses and whole grain foods, as well as lean proteins and lower fat dairy products, with limited foods that are high in saturated fat, sugar and salt. Adhering to these dietary guidelines promotes better outcomes for both mother and baby.
- Many women, especially those in lower‐income households or facing food insecurity, struggle to meet nutritional guidelines due to limited access to nutritious food, higher stress and financial barriers. This is exacerbated by the ongoing cost‐of‐living crisis, which forces reliance on energy‐dense but nutrient‐poor foods.
- Some women, especially those in vulnerable groups, also face challenges in accessing or adhering to recommendations around supplementation (e.g., folic acid). Improving access to supplements and nutritious food is essential to prevent adverse pregnancy outcomes.

- Healthcare providers need appropriate training and resources to effectively address dietary, weight and food safety concerns during pregnancy. Empowering healthcare professionals will help women adopt healthier lifestyles and ensure better maternal and fetal health outcomes, potentially breaking cycles of poor health across generations.

## 11 Conclusions

This briefing paper has described how a healthy, varied diet, supported by appropriate supplementation, can support fetal growth and development, and maternal health during pregnancy. Maternal obesity and poor diets can increase the risk of adverse pregnancy outcomes and complications such as gestational diabetes and preeclampsia, while also contributing to the risk of the offspring developing obesity. These conditions elevate the likelihood of developing chronic diseases, including type 2 diabetes and cardiovascular issues, later in life for both mother and child. In the United Kingdom, nearly one in five pregnant women are classified as living with obesity at their first antenatal appointment (RCOG 2018), highlighting the significant role of pre‐pregnancy weight and healthy diet in shaping maternal and fetal health. Despite this, many women of childbearing age in the United Kingdom continue to consume diets high in saturated fat, salt and sugar, but low in fibre‐rich foods and key micronutrients (OHID 2025). As a consequence, many enter pregnancy with inadequate intakes of key nutrients during pregnancy, particularly iron, folate, vitamin D and iodine.

The recent House of Lords report has called for urgent action in the United Kingdom, emphasising the need for improved nutrition and weight management both before and during pregnancy to ensure better outcomes for mothers and children (House of Lords 2024). Specific population groups, including teenage girls, women with multiple pregnancies, and those from lower socio‐economic backgrounds, face particular nutritional challenges, especially in adhering to supplementation advice and meeting the recommended intake of folic acid and vitamin D. Furthermore, with plant‐based eating becoming increasingly popular to support both population and planetary health (Proveg 2024), there is a need to continue to monitor micronutrient intakes among women, particularly those commonly provided by animal‐based foods—vitamin B12, iodine, DHA and iron. Tailored advice on plant‐based sources and supplementation where needed will help to address these nutritional gaps

Pregnancy presents a unique opportunity to improve both maternal and fetal health, making it vital to identify key intervention points to encourage healthier behaviours. By providing consistent, evidence‐based guidance on diet, supplementation, physical activity and other lifestyle behaviours throughout pregnancy, healthcare professionals can help address nutritional inadequacies, promote better health outcomes and support lasting lifestyle changes. Early, targeted interventions are especially crucial for vulnerable populations, where timely support can significantly improve long‐term maternal and child health.

## Conflicts of Interest

A.M.G. is a member of the Scientific Advisory Panel on Sweeteners supported by the International Sweeteners Association (for which Ulster University has received consultancy/honoraria); A.J.H. is the recipient of a research grant from the Fruit Juice Science Centre. J.T.G. has received research funding from BBSRC, MRC, British Heart Foundation, Clasado Biosciences, Lucozade Ribena Suntory, ARLA Foods Ingredients, Cosun Nutrition Center, Innocent Drinks and the Fruit Juice Science Centre; is a (non‐exec) scientific advisory board member to ZOE and has completed paid consultancy for 6d Sports Nutrition, Science in Sport, The Dairy Council, PepsiCo, Violicom Medical, Tour Racing Ltd. and SVGC. For a full list of disclosures see https://gonzalezjt1.wordpress.com/2024/03/. A.H. and S.A.S. are employees of the British Nutrition Foundation. Funding to support the British Nutrition Foundation's charitable aims and objectives comes from a range of sources including membership, donations and project grants from food producers and manufacturers, retailers and food service companies, contracts with government departments; conferences, publications and training; overseas projects; funding from grant providing bodies, trusts and other charities. Further information about the British Nutrition Foundation's activities, funding and governance can be found at https://www.nutrition.org.uk/aboutbnf/. K.H.H. declares no conflicts of interest.

## Data Availability

The authors have nothing to report.

## Endnotes

^1^
The NHS defines ‘planning a pregnancy’ as undertaking the following behaviours before trying to conceive: (1) starting folic acid supplementation, (2) stopping smoking, (3) cutting down on alcohol, (4) achieving a healthy weight, (5) reviewingmedications, (6) ensuring vaccinations are up to date, (7) reviewing the management of any long term conditions such as diabetes, (8) screening for inherited blood disorders (NHS 2023a).

^2^
The NICE evidence review found no papers using a 5 mg dose and so this recommendation is based on extrapolation from three studies reporting the impact of supplementing with 4 mg/day in high‐risk individuals (NICE 2025). Five milligrams was retained as the advised dose on the basis of availability in UK pharmacies and to align with existing public advice on the NHS website. For more information ‐ : https://www.nice.org.uk/guidance/ng247/evidence/a‐high‐dose‐folic‐acid‐supplementation‐before‐and‐during‐the‐first‐12‐weeks‐of‐pregnancy‐pdf‐13620108493.
